# Mutant MRPS5 affects mitoribosomal accuracy and confers stress‐related behavioral alterations

**DOI:** 10.15252/embr.201846193

**Published:** 2018-09-20

**Authors:** Rashid Akbergenov, Stefan Duscha, Ann‐Kristina Fritz, Reda Juskeviciene, Naoki Oishi, Karen Schmitt, Dimitri Shcherbakov, Youjin Teo, Heithem Boukari, Pietro Freihofer, Patricia Isnard‐Petit, Björn Oettinghaus, Stephan Frank, Kader Thiam, Hubert Rehrauer, Eric Westhof, Jochen Schacht, Anne Eckert, David Wolfer, Erik C Böttger

**Affiliations:** ^1^ Institut für Medizinische Mikrobiologie Universität Zürich Zürich Switzerland; ^2^ Anatomisches Institut Universität Zürich Zürich Switzerland; ^3^ Institut für Bewegungswissenschaften und Sport ETH Zürich Zürich Switzerland; ^4^ Department of Otolaryngology Kresge Hearing Research Institute University of Michigan Ann Arbor MI USA; ^5^ Transfaculty Research Platform Molecular and Cognitive Neurosciences Universitäre Psychiatrische Kliniken Basel Basel Switzerland; ^6^ GenOway Lyon Cedex 07 France; ^7^ Neuro‐ und Ophthalmopathologie Universitätsspital Basel Basel Switzerland; ^8^ Functional Genomics Center Zurich ETH Zürich und Universität Zürich Zürich Switzerland; ^9^ Institut de biologie moléculaire et cellulaire du CNRS Université de Strasbourg Strasbourg France; ^10^ Present address: Department of Otolaryngology – Head and Neck Surgery Keio University School of Medicine Tokyo Japan

**Keywords:** aging, disease, misreading, mitochondria, protein synthesis, Molecular Biology of Disease, Protein Biosynthesis & Quality Control

## Abstract

The 1555 A to G substitution in mitochondrial 12S A‐site rRNA is associated with maternally transmitted deafness of variable penetrance in the absence of otherwise overt disease. Here, we recapitulate the suggested A1555G‐mediated pathomechanism in an experimental model of mitoribosomal mistranslation by directed mutagenesis of mitoribosomal protein MRPS5. We first establish that the ratio of cysteine/methionine incorporation and read‐through of mtDNA‐encoded MT‐CO1 protein constitute reliable measures of mitoribosomal misreading. Next, we demonstrate that human HEK293 cells expressing mutant V336Y MRPS5 show increased mitoribosomal mistranslation. As for immortalized lymphocytes of individuals with the pathogenic A1555G mutation, we find little changes in the transcriptome of mutant V336Y MRPS5 HEK cells, except for a coordinated upregulation of transcripts for cytoplasmic ribosomal proteins. Homozygous knock‐in mutant *Mrps5* V338Y mice show impaired mitochondrial function and a phenotype composed of enhanced susceptibility to noise‐induced hearing damage and anxiety‐related behavioral alterations. The experimental data in V338Y mutant mice point to a key role of mitochondrial translation and function in stress‐related behavioral and physiological adaptations.

## Introduction

Mitochondria are multi‐functional organelles involved in oxidative metabolism, cellular homeostasis, and signal transduction. They are the primary energy‐providing system in eukaryotic cells by accommodating the enzymatic machinery of oxidative phosphorylation for ATP production. The mitochondrial oxidative phosphorylation system is made up of more than 100 different proteins, 13 of which are encoded by mitochondrial DNA and all the others by nuclear genes 1. Mitochondrial DNA is maternally inherited, and the human mtDNA encodes in addition to the 13 proteins of the respiratory electron transport chain (ETC) for 22 tRNA and two ribosomal RNA genes required for their translation 2, 3.

Mitochondria are of endosymbiotic origin 4, and the mitochondrial ribosomes are more closely related to bacterial ribosomes than to eukaryotic cytosolic ribosomes 5. Mitochondrial protein synthesis allows for the translation of the 13 respiratory subunits encoded by the mitochondrial genome 6. All of the more than 1,000 other proteins of the mammalian mitochondrial proteome are synthesized by the cytosolic ribosome 7, 8, 9, 10.

Classical mitochondrial diseases result from mutations in mtDNA or nuclear genes that disrupt mitochondrial function 11, 12. Back in 1993, a pivotal study established that the nucleotide 1555 A to G substitution in the mitochondrial 12S rRNA gene predisposes to maternally transmitted, non‐syndromic sensorineural deafness 13. The A1555G mutation produces a clinical phenotype that may range from normal hearing to progressive hearing loss or severe congenital deafness 14. Early biochemical investigations of the A1555G mutation using transmitochondrial cell lines pointed to a role for mitochondrial protein synthesis 15, but the molecular mechanisms involved remained obscure 16, 17, 18. In the absence of experimental models of mtDNA manipulation, we have previously used bacterial genetics to reconstruct the corresponding rRNA substitution in bacterial ribosomes. These studies suggested that the pathogenic A1555G mutation affects ribosomal accuracy 19. The postulated mechanism, however, was difficult to reconcile with the significant and diverse pathologies associated with mutations in nuclear‐encoded mitochondrial aminoacyl‐tRNA synthetases 20, 21, 22 and the apparent absence of A1555G‐associated disease in non‐cochlear tissues. Here, to address this question, we mutated the nuclear‐encoded mitochondrial ribosomal protein MRPS5 to result in an experimental model of mitoribosomal mistranslation.

## Results

### Identification of a potentially misreading mutation in mitoribosomal protein MRPS5 and in homologous bacterial ribosomal protein RpsE

The amino acid replacement S200Y in yeast cytosolic ribosomal protein Rps2 (uS5) is a ribosomal ambiguity mutation (*ram*) that confers misreading in yeast cytosolic ribosomes 23. We modeled the corresponding amino acid replacement on available crystal structures of the homologous mitochondrial protein MRPS5 (uS5m) and homologous bacterial protein RpsE (uS5) 24, 25. Modeling indicates that replacement of the corresponding native amino acid with the large aromatic tyrosine residue in both mitochondrial MRPS5 (human *MRPS5* V336Y) and bacterial RpsE (*Escherichia coli rpsE* A127Y) would result in steric hindrance within the protein C‐terminal domain, similar to what is observed for S200Y in the yeast Rps2 protein (Appendix Fig S1).

### RpsE A127Y is a misreading mutation in bacterial ribosomes

**Figure 1:**
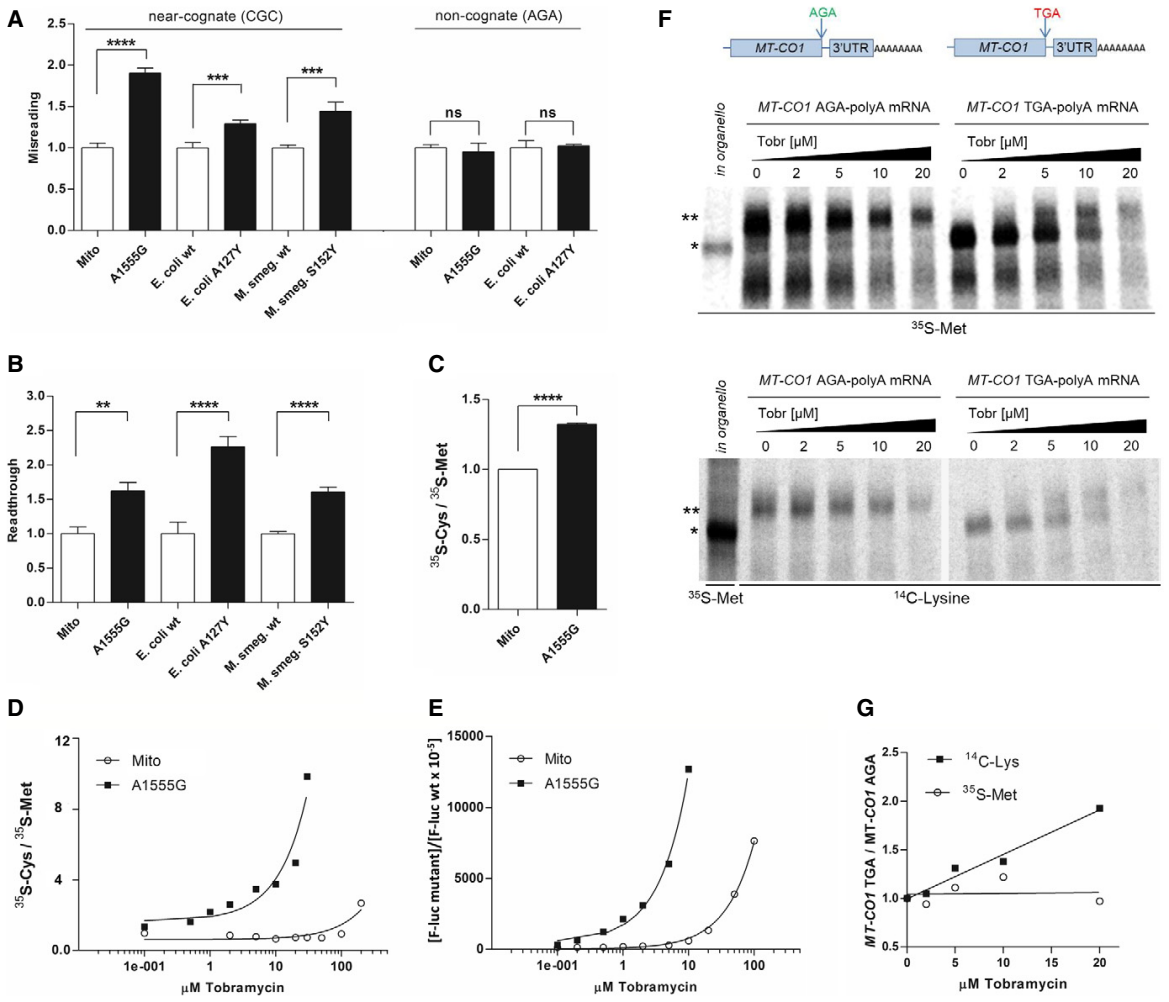
Determination of translational accuracy in cell‐free translation assays A, BMisreading (A) and read‐through (B) were measured by dual luciferase gain‐of‐function assays using wild‐type mitohybrid ribosomes (*n* = 8 misreading, *n* = 4 read‐through), mutant A1555G mitohybrid ribosomes (*n* = 8 misreading, *n* = 4 read‐through), *E. coli* wild‐type ribosomes (*n* = 9), *E. coli* mutant A127Y ribosomes (*n* = 9), *M. smegmatis* merodiploid wild‐type ribosomes (*n* = 9), and *M. smegmatis* merodiploid mutant S152Y ribosomes (*n* = 9). Mistranslation was determined by assessing the misreading at His245 (CAC codon) of the F‐luc gene that was replaced by the near‐cognate codon CGC or the non‐cognate codon AGA, both encoding for Arg. To determine read‐through, the Asp357 (GAC codon) of the F‐luc gene was replaced by the nonsense TGA stop codon. Results are derived by calculating mutant hFluc/hRluc activity related to wild‐type hFluc/hRluc activity. Wild‐type samples were set as 1, error bars indicate SEM; **P < 0.01, ***P < 0.005, ****P < 0.001, ns, not significant (Student's *t*‐test).CRatio of ^35^S‐cysteine/^35^S‐methionine incorporation by *in vitro* translation using wild‐type mitohybrid ribosomes, mutant A1555G mitohybrid ribosomes, and *MT‐CO1* mRNA. Following immunoprecipitation, the MT‐CO1 band was quantified by autoradiography, and wild‐type mitohybrid ribosomes were set as 1 (*n* = 3), error bars indicate SEM; ****P < 0.001 (Student's *t*‐test). Products of immunoprecipitation as resolved by SDS–PAGE are shown exemplarily in Appendix Fig S2E.DRatio of ^35^S‐cysteine/^35^S‐methionine incorporation by *in vitro* translation in the presence of tobramycin using wild‐type mitohybrid ribosomes, mutant A1555G mitohybrid ribosomes, and *MT‐CO1* mRNA; the MT‐CO1 band was quantified by autoradiography.EMisreading of wild‐type mitohybrid ribosomes and mutant A1555G mitohybrid ribosomes in the presence of tobramycin. Misreading was assessed by dual luciferase assay, and results derived by calculating mutant hFluc/hRluc activity are related to wild‐type hFluc/hRluc activity.F
*In vitro* translation of *MT‐CO1*‐TGA mRNA and *MT‐CO1*‐AGA mRNA using mutant A1555G mitohybrid ribosomes in the presence of tobramycin (0–20 μM). Autoradiography of immunoprecipitated MT‐CO1 proteins; top: ^35^S‐Met labeling, bottom: ^14^C‐Lys labeling. *In organello*‐translated ^35^S‐Met‐labeled MT‐CO1 was used as a marker. * MT‐CO1, ** MT‐CO1 extended by read‐through; *MT‐CO1* AGA‐polyA and *MT‐CO1* TGA‐polyA constructs used for *in vitro* translation are schematized.GRatio of ^14^C‐Lys/^35^S‐Met‐labeled immunoprecipitated MT‐CO1 proteins. *In vitro* translation using *MT‐CO1*‐TGA mRNA, *MT‐CO1*‐AGA mRNA, and mutant A1555G mitohybrid ribosomes in the presence of tobramycin (0–20 μM). The corresponding MT‐CO1 band was quantified by autoradiography.

To study whether the mutation *rpsE* A127Y is a *ram* mutation in bacterial ribosomes, we generated bacterial mutants by introducing the corresponding mutation into *E. coli* (*rpsE* A127Y) and *Mycobacterium smegmatis* (*rpsE* S152Y) (Appendix Fig S1). We determined ribosomal misreading and read‐through in cell‐free translation assays using dual luciferase gain‐of‐function reporters. In addition, we included bacterial mitohybrid ribosomes with and without the pathogenic A1555G mutation in the analysis. Reconstructions of mitochondrial 12S rRNA in bacterial ribosomes, with the bacterial decoding A‐site in rRNA helix 44 replaced by the corresponding mitochondrial sequence, have established that the A1555G mutation affects translation accuracy 19. *E. coli rpsE* A127Y, *M. smegmatis rpsE* S152Y, and bacterial mitohybrid ribosomes with the pathogenic A1555G mutation all showed significantly increased levels of misreading of near‐cognate codons and read‐through in comparison with their respective controls (Fig 1A and B).

### Ratio of cysteine/methionine incorporation and increased read‐through upon translation of MT‐CO1 mRNA are markers for ribosomal misreading

Mitochondrial ribosomes are neither suitable for cell‐free translation assays, nor is it technically feasible to introduce reporter genes to probe mitoribosomal accuracy, given the current limitations in genetic manipulation of mitochondria of higher eukaryotes 26.

Amino acid sequence analysis of the 13 mtDNA‐encoded human proteins (NCBI, JO1415.2) revealed that the mitochondrial cytochrome oxidase subunit I MT‐CO1 (COX1) protein contains a single cysteine and 32 methionines. Additionally, the nucleic acid sequence encoding MT‐CO1 shows 96 near‐cognate codons for cysteine and 48 near‐cognate codons for methionine. This yields a ratio of near‐cognate/cognate codons for cysteine versus near‐cognate/cognate codons for methionine of 64.0 [(96/1)/(48/32); for calculation of cognate and near‐cognate frequencies, see Appendix Table S1]. Based on these calculations, we hypothesized that misreading would increase the ratio of cysteine/methionine incorporation and decided to assess ribosomal accuracy by determining the amount of cysteine versus methionine incorporation in MT‐CO1 protein.

We used bacterial A1555G mutant and wild‐type mitohybrid ribosomes to demonstrate that experimental determination of cysteine/methionine incorporation in MT‐CO1 can be used to assess misreading. The *MT‐CO1* sequence adapted to bacterial codon usage resulted in a change in the near‐cognate/cognate codon ratio for cysteine versus methionine to 86.7 [(130/1)/(48/32); the ratio is different from the mitochondrial ratio because bacteria have fewer near‐cognate methionine codons]. The ratio of cysteine/methionine incorporated upon *in vitro* translation of *MT‐CO1* mRNA was significantly increased in the mutant A1555G mitohybrid ribosomes as compared to wild‐type mitohybrid ribosomes (Fig 1C). Further support for this observation was provided by the finding that treatment with the aminoglycoside tobramycin increased misreading in a similar manner when assessing MT‐CO1 cysteine/methionine incorporation or when using a luciferase gain‐of‐function assay, for both A1555G mitohybrid ribosomes and wild‐type mitohybrid ribosomes (Fig 1D and E; note that the pathogenic A1555G mutation confers hypersusceptibility to tobramycin‐induced misreading 19).

The hungry AGA codon at the 3′ end of the MT‐CO1 protein results in a −1 frameshift on the ribosome, allowing a UAG stop codon to be recognized by mitochondrial release factor mtRF1a 27. We hypothesized that misreading mitochondrial ribosomes should bypass the MT‐CO1 stop signal by read‐through, resulting in an extended protein with a C‐terminal poly‐lysine stretch (no stop codon is present in the *MT‐CO1* 3′ UTR in‐frame or in −1 frame, facilitating translation of the polyA tail). We first validated this assay using bacterial A1555G mitohybrid ribosomes because of their hypersusceptibility to aminoglycosides 19. Using the *MT‐CO1* sequence adapted to bacterial codon usage, two synthetic mRNAs were generated—one *MT‐CO1* mRNA with the native AGA codon (*MT‐CO1* AGA, AGA encoding arginine in bacterial translation) and a second *MT‐CO1* mRNA with the AGA codon replaced by a UGA stop codon (*MT‐CO1* TGA). Both constructs carry the 3′ UTR of *MT‐CO1* (69 nt) followed by a polyA tail of 51 nucleotides. In the absence of aminoglycosides, A1555G hybrid ribosomes produced a 57.1 kD‐sized protein corresponding to the native MT‐CO1 protein when translating *MT‐CO1* UGA mRNA and an extended MT‐CO1 62.1 kD‐sized protein when translating *MT‐CO1* AGA mRNA (Fig 1F). Two effects were observed in the presence of increasing amounts of aminoglycoside. First, protein synthesis became inhibited due to the inhibitory effect of tobramycin on ribosomal translocation. Second, increasing amounts of the extended 62.1 kD MT‐CO1 protein were produced for the *MT‐CO1* UGA mRNA due to tobramycin‐induced UGA stop codon read‐through, 3′ UTR translation, and synthesis of a poly‐lysine stretch (Fig 1F). In further support of our conclusion, *in vitro* translation in the presence of ^14^C‐lysine showed that the ratio of ^14^C‐lysine/^35^S‐methionine incorporation increased in the presence of tobramycin (Fig 1G), as expected when aminoglycoside‐induced read‐through results in the synthesis of a poly‐lysine stretch. No extended MT‐CO1 protein was synthesized in the presence of tobramycin upon translation of *MT‐CO1* mRNA with a TAA stop codon lacking 3′ UTR and polyA tail (Appendix Fig S2A).

Collectively, these data demonstrate that both the ratio of cysteine/methionine incorporation and the increased read‐through upon translation of MT‐CO1 mRNA constitute valid markers for ribosomal misreading.

### Mutation V336Y in MRPS5 confers mitoribosomal misreading in *in organello* translation

We next generated stably transfected human HEK cells of mutant V336Y *MRPS5* and wild‐type *MRPS5* controls. We found evidence that the transgene‐encoded MRPS5 protein localizes to the mitochondrial fraction using myc‐tagged MRPS5 (Appendix Fig S2B). A GFP reporter was added downstream of *MRPS5* via an internal ribosomal entry site (IRES) to facilitate monitoring of transgene expression. V336Y mutants and wild‐type control transfectants showed similar GFP fluorescence (Appendix Fig S2C). We quantified *MRPS5* mRNA expression by RT–qPCR to demonstrate that transfected V336Y mutant *MRPS5* and transfected wild‐type *MRPS5* produce similar levels of *MRPS5* mRNA (Appendix Fig S2D).

**Figure 2:**
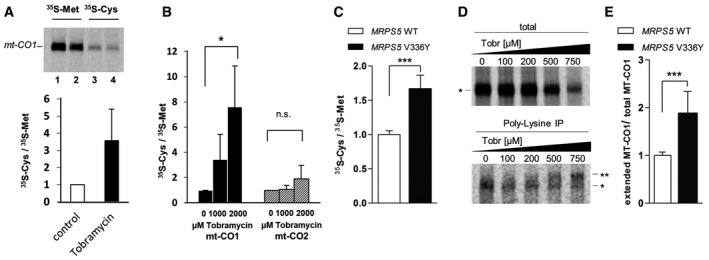
Assessment of mitoribosomal misreading in *in organello* translation Autoradiography of *in organello* mitochondrial translation products derived from HEK293 wild‐type cells in the presence of tobramycin, incorporation of ^35^S‐Met and ^35^S‐Cys in MT‐CO1 protein. Following *in organello* translation proteins were immunoprecipitated and analyzed by autoradiography. Lane 1, 3: controls; lanes 2, 4: *in organello* translation in the presence of 1,000 μM tobramycin. MT‐CO1 bands were quantified and ratio of ^35^S‐Cys/^35^S‐Met calculated, and the ^35^S‐Cys/^35^S‐Met ratio in the absence of tobramycin was set as 1 (*n* = 3), error bars indicate SEM.Ratio of ^35^S‐Cys/^35^S‐Met‐labeled MT‐CO1 and MT‐CO2 proteins synthesized in *in organello* translation and effect of tobramycin. Following *in organello* translation in the presence of tobramycin (1,000 μM, 2,000 μM), proteins were immunoprecipitated and analyzed by autoradiography. MT‐CO1 and MT‐CO2 bands were quantified, and ratio of ^35^S‐Cys/^35^S‐Met incorporation was calculated (*n* = 3), error bars indicate SEM; *P < 0.05 (Student's *t*‐test), ns, not significant.Ratio of ^35^S‐Cys/^35^S‐Met‐labeled MT‐CO1 protein synthesized in *in organello* translation using mitochondria from HEK293 MRPS5 wild‐type cells and HEK MRPS5 mutant V336Y cells. Following *in organello* translation, proteins were immunoprecipitated and analyzed by autoradiography. MT‐CO1 bands quantified and ratios calculated. The ratio of the MRPS5 wild‐type was set as 1 (*n* = 8 clones), error bars indicate SD; ***P < 0.0001 (Student's *t*‐test).Autoradiography of *in organello* mitochondrial translation products derived from HEK293 *MRPS5* wild‐type cells in the presence of tobramycin (0–750 μM). Proteins were ^35^S‐Met‐labeled and loaded either directly on SDS–PAGE (total, top) or following poly‐lysine immunoprecipitation (bottom). * MT‐CO1 and ** MT‐CO1 with poly‐lysine extension.Quantification of MT‐CO1 proteins in HEK293 *MRPS5* wild‐type cells and HEK293 *MRPS5* mutant V336Y cells. *In organello* mitochondrial translation in the presence of ^35^S‐Met and 750 μM tobramycin. Proteins were loaded either directly on SDS–PAGE (total) or following poly‐lysine immunoprecipitation and analyzed by autoradiography. For total protein samples, the MT‐CO1 band was quantified, for poly‐lysine immunoprecipitated samples, the extended MT‐CO1 band was quantified and the ratio of extended MT‐CO1/total MT‐CO1 was calculated. The ratio of *MRPS5* wild‐type was set as 1 (*MRPS5* wild‐type, *n* = 5 clones, *MRPS5* V336Y, *n* = 7 clones), error bars indicate SD; ***P < 0.0001 (Student's *t*‐test).

To investigate the accuracy of mitochondrial translation, we used *in organello* translation and determined the ratio of cysteine/methionine incorporation in MT‐CO1 protein. *In organello* translation in the presence of the misreading agent tobramycin resulted in a significantly increased ratio of cysteine/methionine incorporation in MT‐CO1 protein (Fig 2A). Compared to MT‐CO1 with a ratio of 64.0 for near‐cognate/cognate codons for cysteine versus methionine, this ratio is only 3.8 for the mtDNA‐encoded cytochrome oxidase subunit 2 MT‐CO2 (near‐cognate/cognate codons for cysteine versus near‐cognate/cognate codons for methionine: 29/3 versus 26/10, see Appendix Table S1). We thus predicted that compared to MT‐CO1, *in organello* translation in the presence of the misreading agent tobramycin should not grossly affect the ratio of cysteine/methionine incorporation in MT‐CO2. As shown in Fig 2B, treatment with tobramycin resulted in a dose‐dependent increase in the ratio of cysteine/methionine incorporation in MT‐CO1 protein, but did not measurably affect this ratio in MT‐CO2 protein. We next studied the ratio of cysteine/methionine incorporation in MT‐CO1 protein in mutant V336Y MRPS5 and wild‐type control transfectants. In comparison with the wild‐type control mutant, V336Y *MRPS5* transfectants showed a significantly increased ratio of cysteine/methionine incorporation in MT‐CO1 protein (Fig 2C).

Immunoprecipitation with anti‐lysine antibodies was used to study read‐through of MT‐CO1 in mitochondrial *in organello* translation. In the presence of increasing tobramycin concentrations, increasing amounts of extended MT‐CO1 protein were produced (Fig 2D). Compared to wild‐type *MRPS5* transfected cells, mitochondrial extracts from mutant V336Y cells showed significantly increased read‐through of the MT‐CO1 protein (Fig 2E).

Together, and consistent with our modeling predictions, these findings demonstrate that the *MRPS5* V336Y mutation confers mitoribosomal misreading.

### Effects of MRPS5 V336Y‐mediated mitoribosomal misreading in HEK293 cells

**Figure 3:**
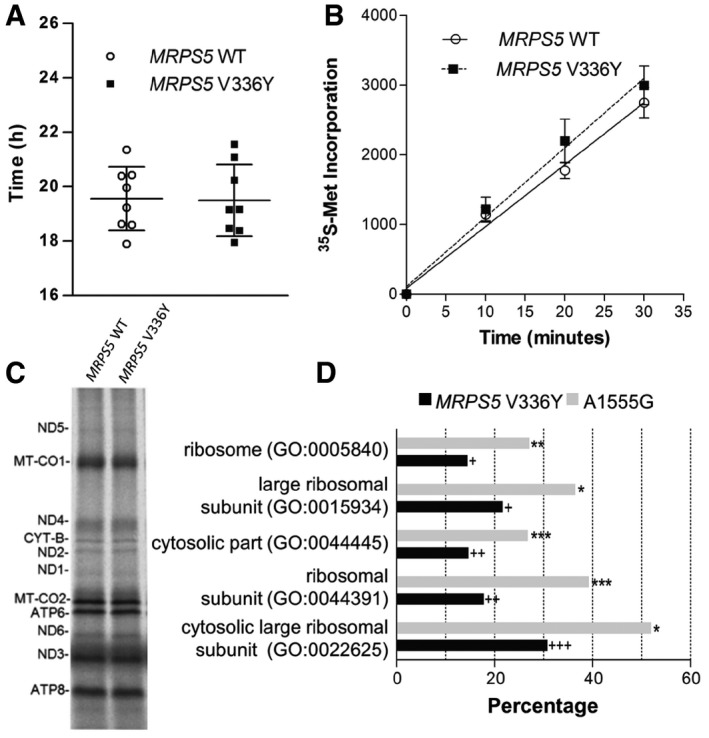
Analysis of HEK293 mutant *MRPS5* V336Y and HEK293 *MRPS5* wild‐type cells Generation time (*n* = 8 clones, ± SD).Whole cell *in vivo* mitochondrial translation as determined by ^35^S‐Met incorporation (*n* = 4 clones, ± SEM).Autoradiography of *in organello* translation using ^35^S‐Met labeling.Top five GO terms (cellular component) associated with genes upregulated in HEK293 mutant *MRPS5* V336Y compared to HEK293 wild‐type *MRPS5*. Corresponding GO terms associated with genes upregulated in mutant *MT‐RNR1* A1555G lymphocytes (Hollis *et al*, 2015) were plotted for comparison. The bars represent the percentage of genes that map to a GO term and which are upregulated. Fisher's exact *t*‐test, ^+^ *P <* 10^−6^, ^++^ *P <* 10^−7^, ^+++^ *P <* 10^−8^ (*MRPS5* V336Y *n* = 4, *MRPS5* wild‐type *n* = 6); *P < 10^−30^, **P < 10^−40^, ***P < 10^−50^ (A1555G *n* = 28, wild‐type *n* = 15).

Measurements of ATP levels, mitochondrial mass, mtDNA/nuclear DNA ratio, and oxygen consumption showed no significant differences between wild‐type *MRPS5*‐transfected and mutant V336Y *MRPS5*‐transfected cells (Appendix Fig S3A–D). Measurements of generation time and quantitative (^35^S‐Met incorporation) and qualitative (SDS–PAGE of ^35^S‐Met‐labeled proteins) assessments of mitochondrial translation (Fig 3A–C) also showed no differences. We corroborated the finding that generation time and protein synthesis efficacy were unaffected by using the corresponding mutation in the bacterial system (*E. coli*—*rpsE* A127Y, *M. smegmatis—rspE* S152Y; see Appendix Fig S3E and F). These data indicate that, despite conferring misreading, the homologous mutations in ribosomal proteins *MRPS5* and *rpsE* do not grossly affect efficiency of protein synthesis in phylogenetically related translation systems. Together, these data demonstrate that the mitoribosomal misreading mutation *MRPS5* V336Y little affects HEK293 cells.

In comparison with wild‐type MRPS5‐transfected cells, transcriptomic analysis of the V336Y mutants by RNA sequencing revealed little differences, except for an overexpression of cytoplasmic ribosomal proteins. The top five affected GO terms were all cytoplasmic ribosome‐related (GO term ribosome, adjusted *P*‐value 2.46 × 10^−4^; see Fig 3D).

### Mutation V338Y in MRPS5 affects ETC function in brain cells *in vivo*

Next, we constructed homozygous knock‐in mutant *Mrps5* mice (*Mrps5* ^V338Y/V338Y^; in mice, the homologous position in *Mrps5* is V338Y, see Appendix Fig S1) using a FLEx strategy; for further details, see Materials and Methods and Appendix Fig S4. Mutant *Mrps5* V338Y mice are viable, born at the expected frequencies, have normal physical appearance, and show no overt signs of disease.

**Figure 4:**
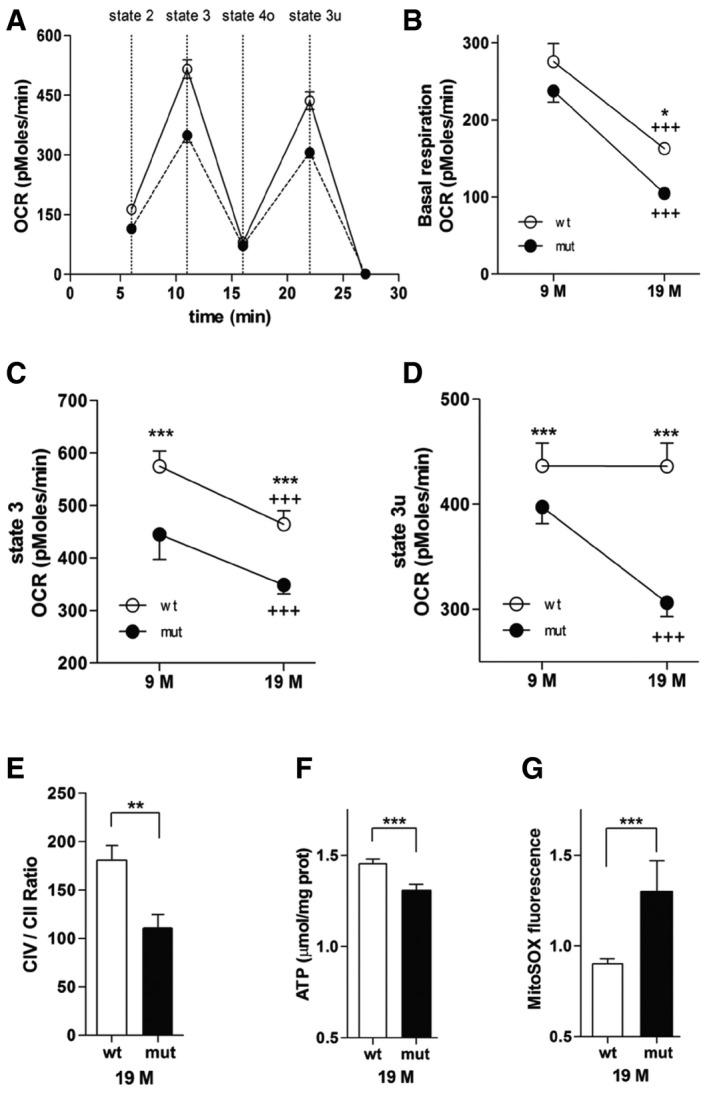
Mutation‐related mitochondrial function in brain A–DOxygen consumption rate (OCR) was measured in freshly isolated mitochondria from wild‐type and mutant mouse cortex. (A) OCR in brain mitochondria from 19‐month‐old mutant mice (black circle) compared with OCR from wild‐type mice (white circle). The sequential injection of mitochondrial substrates and inhibitors is indicated by dotted lines (see details in the Materials and Methods section). Values corresponding to the different respiratory states are shown. (B) Baseline OCR, effect of age (*P* < 0.0001), and genotype (*P* < 0.05). (C) State 3 OCR (after addition of ADP), effect of age (*P* < 0.001), and genotype (*P* < 0.0001). (D) State 3 uncoupled OCR (state 3u after addition of FCCP), effect of genotype (*P* < 0.0001), and age–genotype interaction (*P* < 0.0001).ERatio of mitochondrial complex IV/complex II activities.FATP levels.GSuperoxide anion radicals.Data information: Panels (A–D) are represented as mean ± SEM (*n* = 10–12 replicates of five to six animals per group); *P < 0.05, ***P < 0.0001 (wild‐type versus mutant); ^+++^ *P* < 0.0001 (19 months versus 9 months); post hoc test followed by two‐way ANOVA significance test. Panels (E–G) are represented as mean ± SD (*n* = 5–6); **P < 0.001, ***P < 0.0001 (wild‐type versus mutant) (Student's *t*‐test).

We evaluated the capacity of the oxidative phosphorylation system in brain cortical mitochondria of both mature (9‐month‐old) and aged (19‐month‐old) *Mrps5* ^V338Y/V338Y^ and wild‐type control mice using the Seahorse XF24 flux analyzer system (Fig 4A–D). Total oxygen consumption rate (OCR) was significantly reduced in the mitochondria of 9‐ and 19‐month‐old *Mrps5* V338Y mice compared to mitochondria of age‐matched wild‐type mice. Specifically, basal activity (state 2) and maximal respiration (state 3, which measures the capacity of mitochondria to metabolize oxygen in the presence of the ATP synthase substrate ADP) showed an age‐ and mutation‐related decline (Fig 4B and C). In addition, respiration in the absence of a proton gradient (uncoupled state 3u following injection of FCCP) was significantly impaired in the mutant mice (Fig 4D).

Deterioration of mitochondrial function is often related to an age‐dependent decrease of ATP and increased levels of ROS as a result of ETC leakage, prompting us to examine ATP and ROS levels in the mitochondria of 19‐month‐old animals, in addition to measurements of complex II and complex IV activities. In contrast to complex II which is exclusively encoded by nuclear genes, complex IV is encoded by both nuclear and mitochondrial genes. Thus, comparing complex IV activity to that of complex II (complex IV/complex II ratio) highlights the impact of the *Mrps5* V338Y mutation. Compared to wild‐type control mice, the complex IV/complex II ratio was significantly reduced in cortical mitochondria from mutant mice (Fig 4E). Consistent with the finding of a mutation‐related decline in coupling efficiency of ETC, we observed reduced ATP levels and increased amounts of mitochondrial reactive oxygen species (mtROS) in mitochondria of *Mrps5* ^V338Y/V338Y^ mice (Fig 4F and G).

### Mutant MRPS5 mice show age‐related and stress‐induced behavioral alterations

Mutant *Mrps5* ^V338Y/V338Y^ mice and littermates did not present any overt difference in spontaneous behavior. To assess a large spectrum of more subtle behavioral phenotypes, we subjected *Mrps5* ^V338Y/V338Y^ mice and their littermates to a comprehensive battery of tests for changes in learning and memory, species‐typical behavior, anxiety, exploration, and sensorimotor function. We studied mice at the age of 9 and 19 months to detect early‐onset and age‐related phenotypes; selected tests were also recorded at 14 months of age.

Locomotor activities in the home cage, in the large open field, during light–dark transition, and in the elevated O‐maze were affected neither by genotype nor by age (Appendix Fig S5A–D). Swim speed in the water‐maze, performance on the rotarod, grip test, hot plate test, and acoustic startle (Appendix Fig S5E–H) did not show any mutational effect on sensorimotor function. Responses in the acoustic startle were reduced in 19‐month‐old mice, most likely due to the age‐related hearing loss typically seen in C57BL/6 mice 28.

**Figure 5:**
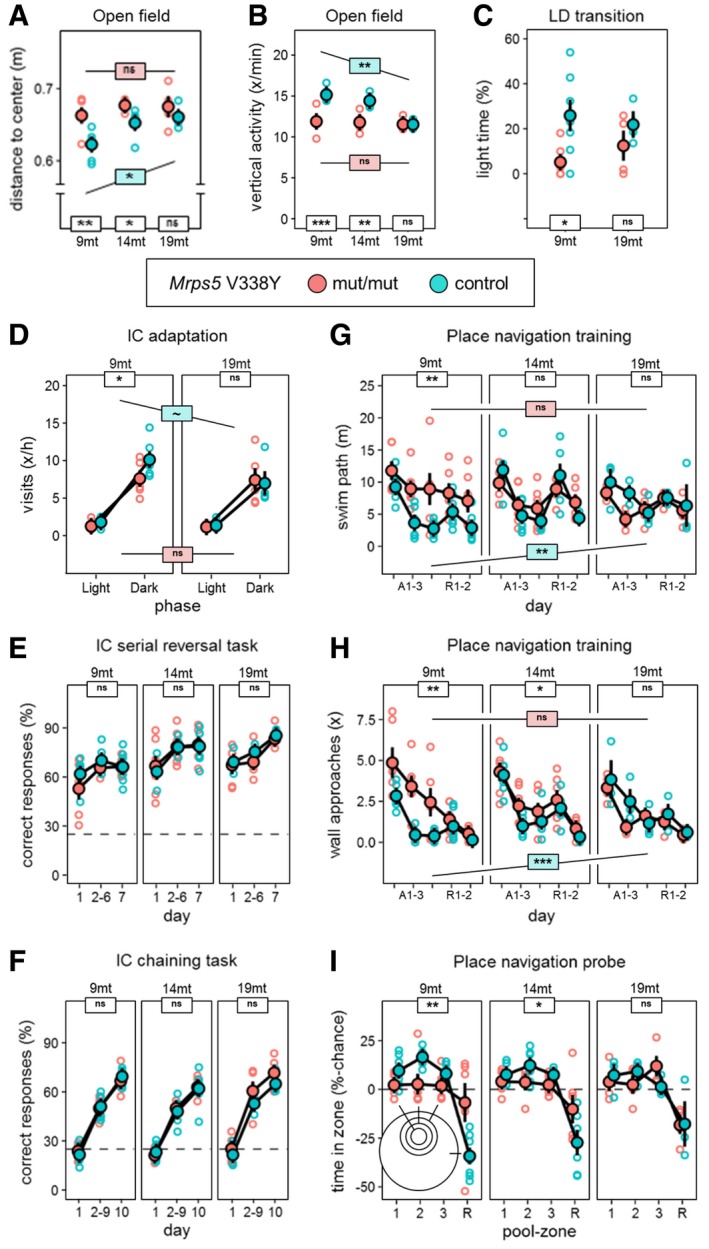
Exploration and anxiety, home cage learning, and place learning in the water‐maze Average distance to center as measure of anxiety in the open field (ANOVA: genotype *F*_1,24_ = 16.24, *P* = 0.0005, age *F*_2,24_ = 5.217, *P* = 0.0131). Mutants show increased anxiety levels at 9 and 14 months.Estimated frequency of rearing activity as measure of exploratory drive in the open field (ANOVA: genotype *F*_1,24_ = 39.40, *P* < 0.0001, age × genotype *F*_2,26_ = 8.204, *P* = 0.0017). Mutants showed little exploratory activity at 9 and 14 months.Light–dark transition test, percentage of time spent in the bright compartment (ANOVA: genotype *F*_1,16_ = 7.393, *P* = 0.0152). Reduced scores at 9‐month‐old animals indicate increased anxiety.Exploratory corner visits in dark and light phase during adaptation to the IntelliCage (ANOVA: phase *F*_1,18_ = 617.2, *P* < 0.0001). Corner visits during the dark phase as a correlate of exploratory activity showed premature reduction in 9‐month‐old animals.Learning in the hippocampus‐dependent IntelliCage serial reversal task (ANOVA: day *F*_2,56_ = 26.71, *P* < 0.0001). Scores provided no evidence for a mutation effect on learning rate or performance.Learning in the hippocampus‐dependent IntelliCage chaining task (ANOVA: day *F*_2,54_ = 450.1, *P* < 0.0001). Scores provided no evidence for a mutation effect on learning rate or performance.Water‐maze place navigation, swim path during acquisition, and reversal (ANOVA: day *F*_4,104_ = 21.14, *P* < 0.0001, genotype *F*_1,26_ = 13.11, *P* = 0.0013, age × genotype *F*_2,26_ = 8.204, *P* = 0.0017). All groups learned: Controls showed reduced performance with increasing age, and mutants showed poor performance already at 9 and 14 months.Number of wall approaches as indicator of poor coping with the test and non‐spatial strategy choice (ANOVA: genotype *F*_1,26_ = 18.31, *P* = 0.0002, age *F*_2,26_ = 4.486, *P* = 0.0212, age × genotype *F*_2,26_ = 7.068, *P* = 0.0035). Number of wall approaches increased with age in controls and was prematurely increased in mutants at 9 and 14 months.Water‐maze probe trial, spatial retention score for target zones 1–3 comprising 5, 10, and 10% of the pool surface versus remaining 75% of surface (ANOVA: zone *F*_3,78_ = 36.04, *P* < 0.0001, genotype × zone *F*_3,78_ = 7.807, *P* = 0.0001). Spatial selectivity declined with age in controls and was prematurely reduced in mutants at 9 and 14 months.Data information: Graphs show mean and SEM. Post hoc FDR corrected, ***P < 0.001, **P < 0.01, *P < 0.05, ~*P* < 0.1, ns *P* ≥ 0.1. White boxes = *t*‐test for genotype within age cohorts, colored boxes = ANOVA age effects contrasting between genotypes. 34 mice, *n* = 17 per genotype, *n* = 9–13 per age cohort.

To evaluate anxiety‐related and exploratory behavior, we tested mice in the large open field, light–dark transition, elevated O‐maze, and IntelliCage. In the large open field, littermate controls showed an age‐dependent increase in center avoidance and a decrease in rearing activity from 9 to 19 months of age. In contrast, *Mrps5* ^V338Y/V338Y^ animals showed pronounced center avoidance and low rearing activity at all ages, with 9‐month‐old *Mrps5* ^V338Y/V338Y^ mice already resembling aged animals (Fig 5A and B). Area explored in the large open field (Appendix Fig S5I) and avoidance of the brightly illuminated chamber in the light–dark transition test (Fig 5C) showed similar age dependency, with *Mrps5* ^V338Y/V338Y^ being less explorative than controls at the age of 9 months but not at the age of 19 months. On the elevated O‐maze, responses of *Mrps5* ^V338Y/V338Y^ animals were statistically indistinguishable from those of controls even though on average they spent slightly less time in the open arms (Appendix Fig S5J). Explorative analysis of individual cohorts during the free adaptation stage in IntelliCage revealed decreased activity for *Mrps5* ^V338Y/V338Y^ mice at 9 months of age (Fig 5D).

Learning and memory were evaluated in the T‐maze spontaneous alternation test and the IntelliCage and compared to performance in the more adverse environment of the water‐maze place and cue navigation tasks. Performance in the T‐maze spontaneous alternation test (Appendix Fig S5K), and learning in the serial reversal (Fig 5E) and chaining (Fig 5F) tasks in IntelliCage were not affected by genotype. In the water‐maze place navigation task, the performance during acquisition and reversal training worsened significantly in the controls with aging. In contrast, *Mrps5* ^V338Y/V338Y^ mice performed poorly already at young age and were significantly impaired compared to the controls at 9 months of age (Fig 5G). The poor escape of the *Mrps5* mutants in the place navigation task was accompanied by an increased number of wall approaches, again with 9‐month‐old mutants earning scores similar to those of aged animals (Fig 5H). In the probe trial (Fig 5I), *Mrps5* mutants showed overall significantly reduced preference for the trained target zone. Consistent with an age‐dependent phenotype of the mutation, a significant zone × genotype interaction was observed for the 9‐ and 14‐month‐old cohorts, but not for the 19‐month‐old animals. The cue navigation protocol in the water‐maze showed a small overall deficit in *Mrps5* mutant mice (Appendix Fig S5L).

From the above results, we conclude that mutation effects on learning task performance were strongly context‐dependent. While we observed no adverse mutation effect or age‐related performance declines in T‐maze alteration and IntelliCage learning, performance in the water‐maze place navigation task was strongly affected by the mutation in an age‐dependent manner. As the performance of the mutants was only affected in the water‐maze and not in other learning tasks, it is unlikely that this deficit is reflective of a genuine cognitive impairment, also supported by the finding that hippocampus‐dependent burrowing and nesting were unaffected by the mutation (Appendix Fig S5M and N). Rather, as also supported by the results of our tests on anxiety and exploration, water‐maze performance was probably disrupted by an excessive sensitivity of *Mrps5* mutants to the aversive stress conditions associated with this test. In less aversive environments such as the T‐maze or IntelliCage, *Mrps5* mutants were able to learn normally.

Comparative brain histopathological analyses of 9‐month‐old *Mrps5* ^V338Y/V338Y^ mice and age‐matched wild‐type controls did not reveal any difference. In particular, no signs of neurodegeneration were found in the mutant mice.

### Mutation V338Y heightens susceptibility to noise‐related auditory damage

**Figure 6:**
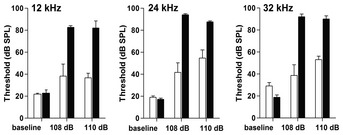
Noise‐induced hearing damage Noise exposure and ABR measurements. Exposure of mice to broadband noise (2–10 kHz) of either 108 or 110 dB SPL resulted in elevations of auditory thresholds. Thresholds in *Mrps5* ^V338Y/V338Y^ mutant mice (black bars) were significantly higher after exposure than thresholds in littermates (open bars); *P* < 0.0001 (two‐way ANOVA for combined 108 and 110 dB data). Frequencies of 12, 24, and 32 kHz were selected for measurement to cover the mid‐frequency to high‐frequency regions of the cochlea. *n* = 6 for baseline littermates; *n* = 6 for baseline mutants; *n* = 3 for all noise exposures. Graphs show mean and SEM.

We selected auditory function *in vivo* as a sensory modality susceptible to environmental stress on mitochondria 29. The non‐invasive procedure of auditory brain stem responses (ABR) records neuronal activity along the auditory pathways and provides information on the functional integrity of the cochlea. *Mrps5* ^V338Y/V338Y^ and control mice had comparable baseline thresholds of around 20 dB sound pressure level (SPL), indicating that the mutation per se did not affect fundamental auditory function (Fig 6). Following an exposure to noise (2–10 kHz broadband at intensities of 108 and 110 dB), ABR thresholds were determined 3 weeks later when damage to the auditory system was stabilized. Exposure to noise resulted in increases in ABR thresholds at 12, 24 and 32 kHz. The noise‐induced auditory damage was significantly more pronounced in *Mrps5* ^V338Y/V338Y^ mice than in the control littermates as evidenced by consistently larger threshold shifts at all three frequencies. Furthermore, while control mice showed a graded response to noise with increasing thresholds from 108 to 110 dB, V338Y mutants demonstrated a ceiling effect already at 108 dB corroborating their heightened susceptibility to noise trauma (Fig 6).

## Discussion

By genetic manipulation of the nuclear‐encoded mitochondrial ribosomal protein MRPS5, we have established the first‐ever reported mammalian model of a mitochondrial *ram* mutant. Following the *in silico* identification of a putative *ram* mutation in mammalian MRPS5 (human V336Y, mouse V338Y), phylogenetic reconstructions provided evidence that the corresponding mutation A127Y in homologous bacterial ribosomal protein *rpsE* (*E. coli*) results in mistranslation as evidenced by increased incorporation of near‐cognate aa‐tRNA species and stop codon read‐through. In the absence of established means to introduce reporter genes into mitochondria of higher eukaryotes, we identified the mtDNA‐encoded MT‐CO1 protein as an endogenous reporter of mitoribosomal misreading. Using bacterial mitohybrid ribosomes with and without the pathogenic A1555G mutation and translating MT‐CO1 mRNA in the absence and presence of the misreading agent tobramycin, we established that both the ratio of cysteine/methionine incorporation and the read‐through in MT‐CO1 protein translation constitute markers of ribosomal misreading. Subsequently, we directly demonstrated by mitochondrial *in organello* translation that the MRPS5 mutation V336Y increases both the ratio of cysteine/methionine incorporation and read‐through of endogenous MT‐CO1, as does treatment with tobramycin.

We found that mutant MRPS5‐mediated mitochondrial mistranslation in homozygous knock‐in *Mrps5* ^V338Y/V338Y^ mice is associated with impaired mitochondrial function in post‐mitotic cells *in vivo*. Brain cortical mitochondria showed a significant reduction in basal respiration and maximal respiratory capacity, decreased ATP levels, and increased formation of ROS. The cognitive performance of mutant *Mrps5* mice was apparently normal under non‐aversive conditions. However, we observed age‐ and stress‐related alterations in three behavioral traits in *Mrps5* V338Y mutant mice: first, increased anxiety responses as evidenced by less time spent in the center of the open field and more time spent in the dark chamber of the light–dark transition test; second, decreased explorative drive as revealed by diminished vertical activity and a smaller area explored in the open field; and third, impaired learning and memory under stressful conditions as shown by poor training and probe trial scores in the water‐maze place navigation tests. The same combination of behavioral differences characterized the transition from adulthood to old age in the wild‐type control mice, reflecting normal behavioral aging. The triad of symptoms observed in *Mrps5* mutant mice—increased vigilance to threat, reduced exploratory drive, and impaired coping with stress—is typical for physiological behavioral aging in humans and rodents 30, 31, 32, 33. Thus, impaired mitochondrial respiration in part phenocopied non‐cognitive age‐associated alterations in brain function. The pathological phenotype of neurological stress intolerance and anxiety‐related behavioral alterations in *Mrps5* ^V338Y/V338Y^ mice points to a key role for mitochondrial function in stress‐associated behavioral adaptations 34, 35, 36.

Auditory studies revealed another compelling example of a mitochondria‐related pathology in the mutants. Auditory thresholds, reflecting the integrity of sensory hair cells and ascending auditory pathways, are normal in V338Y mutant animals compared to littermate controls. This mirrors the normal hearing phenotype of most A1555G patients. The A1555G mutation shows a variable phenotype from lifelong normal hearing to severe hearing loss, pointing to a role for additional genetic and environmental stress factors necessary for expression and exacerbation of a pathological phenotype 37, 38. Notable stressors for the A1555G mutation are aminoglycoside antibiotics whose administration can lead to sudden profound hearing loss 13. The hypersusceptibility to aminoglycoside ototoxicity in A1555G patients is at least in part conferred by increased binding of the drug to its target—rRNA residue 1555 is part of the drug binding pocket in the ribosomal A‐site 19. In the absence of increased binding of aminoglycosides to mutant V338Y ribosomes—the ribosomal protein MRPS5 is not part of the drug binding pocket—we decided to use noise trauma as a particular pertinent stressor because of its traumatic mechanisms impinge on sensory hair cell mitochondria 29. Compared to wild‐type control mice, mutant V338Y mice showed significantly heightened susceptibility to noise trauma. The increased loss of auditory function in *Mrps5* ^V338Y/V338Y^ mutant mice observed upon exposure to noise apparently results from noise‐induced enhanced ROS formation, ATP depletion, and mitochondrial cell death signaling 39, 40, superimposed on an already mutation‐mediated compromised mitochondrial function.

In contrast to several human disease‐causing mutations in mitochondrial tRNAs and nuclear genes encoding mitochondrial aminoacyl‐tRNA synthetases 11, 22, the mutant MRPS5 mice display a remarkable absence of overt pathology *in vivo*. While this surprising observation may reflect a threshold effect of the mutation studied, our findings are supported by the only discrete alterations observed in humans with the mechanistically similar pathogenic A1555G mutation 37. Our results from whole genome transcriptome analyses of the V336Y mutant HEK cells are in agreement with microarray‐based studies in immortalized lymphocytes of individuals with the mitochondrial A1555G mutation which revealed little changes, except for a coordinated upregulation of cytosolic ribosomal proteins, an effect that was suggested as a compensatory mechanism for the mitochondrial dysfunction associated with the A1555G mutation 41. In addition to a possible threshold effect, the most probable explanation for the lack of overt pathology in A1555G patients and MRPS5 V338Y mutant mice may reside in the nature of ribosomal misreading. Disease‐causing mutations in mitochondrial tRNA or aminoacyl‐tRNA synthetases affect a specific tRNA and every molecule thereof with detrimental consequences for translation 22, 42, 43. In contrast, misreading by *ram* mutations affects translational accuracy in a random and stochastic manner, which apparently can be in part tolerated. In addition, while *ram* mutations, such as A1555G or MRPS5 V338Y, may allow for increased rates of near‐cognate misreading, the incorporation of non‐cognate amino acids during translation is prevented by the difference in the free binding energy between cognate and non‐cognate aa‐tRNAs 44.

In conclusion, by mutagenesis of nuclear‐encoded mitochondrial protein MRPS5 we have established a unique model of mitoribosomal misreading that recapitulates the suggested A1555G pathomechanism 15, 16, 17, 18, 19. Evidence is provided that increased mitochondrial misreading is indeed the cause of hearing loss in the A1555G patients. The model reproduces the hearing‐related deficit as well as the absence of overt non‐cochlear pathology that distinguishes A1555G from classical mitochondrial diseases 11, 22. Further, the neurological alterations observed under adverse conditions and the noise‐induced auditory trauma found in MPRS5 mutant mice point to a key role for mitochondrial function in stress‐related behavioral and physiological adaptations.

## Materials and Methods

### Bacterial strains

For generation of merodiploid *M. smegmatis rpsE* S152Y, genomic DNA from *M. smegmatis* served as template for *rpsE* gene amplification. Fusion PCR via complementary overhanging sequences was used for gene construction. The first PCR used 5′ primer fwd1 (5′‐ATA CAA AAC CAG GTC AGG AGA TCG AAT GGC CGA GCA GGC TGG CGC‐3′, native leader sequence of the *rpsE* operon at the 5′ end) and 3′ primer rev1 (5′‐ACT ACT AGT TTA TGC CGA TCC CTC ACG CG‐3′, SpeI ACTAGT recognition site at the 3′ end) to amplify the *rpsE* coding DNA sequence (CDS). The second PCR used plasmid pMIH 45 as template and 5′ primer fwd2 (5′‐ACT AAG CTT CGG TGA CCA CAA CGC GCC CG‐3′) and 3′ primer rev2 (5′‐TCG ATC TCC TGA CCT GGT TTT GTA TCG CAA TTG TCT TGG CCA TTG‐3′) to amplify the hsp60 promotor with a HindIII AAGCTT recognition site at the 5′ end. The two amplicons overlap at the leader sequence and were fused using pfu polymerase (Promega), subcloned into the pGEM^®^‐T Easy vector (Promega), and controlled by DNA sequencing. PCR‐mediated site‐directed mutagenesis was used to introduce the S152Y mutation. The constructs were cloned into integrative vector pMIH using restriction enzymes HindIII and SpeI (Thermo Fisher) resulting in vectors pMIH‐*rpsE‐*wt and pMIH‐*rpsE‐*S152Y, respectively. Vectors pMIH‐*rpsE‐*wt and pMIH‐*rpsE‐*S152Y were electroporated into *M. smegmatis* mc2155 ΔrrnB 46. Single colonies were picked, propagated for further analysis, and checked by PCR and DNA sequence analysis.

For the generation of *E. coli rpsE* A127Y, Oligo λ Red‐mediated recombination was used as described previously 47. In brief, two steps of recombineering were conducted, using *E. coli* MG1655 and oligonucleotides that comprised the A127Y mutation or the wild‐type sequence, respectively. Positive clones were screened by PCR and confirmed by DNA sequencing. The recombinant *E. coli* strains were a kind gift of Diarmaid Hughes and Douglas Huseby, Uppsala University, Sweden.

For growth experiments, bacterial strains were streaked on agar plates and grown at 37°C until single colonies were visible. Single colonies were used to inoculate liquid LB medium. Growth experiments were started at an initial OD_600_ of 0.002 and further incubated at 37°C. OD_600_ was measured every 30 min for *E. coli* and every 3 h for *M. smegmatis*. Signal intensities at time point 0 h were set as one, and growth curves were plotted. Doubling time was calculated as *t*_D_ = ln(2)/slope.

### Cell‐free luciferase translation assays

Purified bacterial 70S ribosomes were used in dual luciferase translation assays as previously described 48, 49. A typical translation reaction (30 μl) contained 0.25 μM 70S ribosomes, 4 μg firefly (F‐luc) and 0.4 μg Renilla (R‐luc) mRNA, 40% (vol/vol) of *M. smegmatis* S100 extract, 200 μM amino acid mixture, 24 units of RiboLock (Thermo Fisher), and 0.4 mg/ml of tRNAs—energy was supplied by the addition of 12 μl of commercial S30 Premix without amino acids (Promega). The reaction mixture was incubated at 37°C for 35 min, stopped on ice, and assayed for F‐luc and R‐luc luciferase activities according to supplier's protocol (Promega). Luminescence was measured using the FLx800 luminometer (BioTek Instruments).

Mistranslation was determined as described previously 50, 51. In brief, to assess misreading His245 (CAC codon) of the F‐luc gene was replaced by the near‐cognate codon CGC or the non‐cognate codon AGA, both encoding for Arg. To determine read‐through, the Asp357 (GAC codon) of the F‐luc gene was replaced by the nonsense TGA stop codon. Arg245F‐luc mRNA, X357TGA F‐luc mRNA, and WT F‐luc mRNA were used in *in vitro* translation reactions, and R‐luc mRNA was used as internal control. Mistranslation was calculated by the ratio of mutant firefly/Renilla luciferase activity to wild‐type firefly/Renilla luciferase activity.

### Cell culture and transfection of HEK293 cells

Human embryonic kidney cells (HEK293, Innoprot) were maintained in complete Dulbecco's modified Eagle's medium (DMEM) (Life Technologies) containing 10% fetal bovine serum (FBS) (Life Technologies), at 37°C in 5% CO_2_.

Plasmid pmouse*Mrps5*‐WT containing the mouse *Mrps5* coding region under the control of the chicken β‐actin promotor and the CMV enhancer (CAGGS promotor) was constructed by Genoway (Lyon, France). In brief, the mouse *Mrps5* wild‐type CDS was synthesized, and restriction sites BamHI and AvrII were introduced at the 5′ and the 3′ ends. Using the BamHI and AvrII restriction sites, the 1,318‐nt‐sized fragment was ligated into an eukaryotic expression vector, which in addition to the CAGGS promotor carries the human growth hormone polyadenylation signal, resulting in pmouse*Mrps5*‐WT.

The plasmid expressing the human *MRPS5* gene was constructed as follows. A cDNA fragment containing human *MRPS5* wild‐type CDS was generated by PCR, using cDNA reverse‐transcribed from total RNA extracted from HEK293 cells. Total RNA was prepared using TRIzol (Life Technologies) and DNase‐treated (DNase I, Life Technologies) by incubation for 15 min at 37°C; 1 μl of 25 mM EDTA was added, and samples were incubated for 10 min at 65°C and stored at −80°C. RNA was reverse‐transcribed using a High Capacity RNA‐to‐cDNA Kit (Life Technologies) according to the manufacturer's instructions. V336Y (GTC→TAC) point mutation was introduced by PCR‐mediated site‐directed mutagenesis, and restriction sites for BamHI and XhoI were introduced at the 5′ and 3′ ends, respectively. The mouse *Mrps5* wild‐type CDS in the pmouse*Mrps5*‐WT vector was replaced with human *MRPS5* wild‐type or human *MRPS5* V336Y using BamHI/XhoI restriction sites, resulting in vectors p*MRPS5*WT and p*MRPS5*V336Y. IRES‐eGFP was amplified from plasmid pLZRSpBMN‐IRES‐eGFP (a kind courtesy of Saule Zhanybekova, University of Basel, Switzerland) and inserted downstream of the *MRPS5* CDS using restriction sites for BstBI and ClaI, resulting in vectors p*MRPS5*WT‐IRES‐eGFP and p*MRPS5*V336Y‐IRES‐eGFP. The hygromycin B resistance cassette was amplified by PCR from vector pGL4.14 (Promega), subcloned into pGEM^®^‐T Easy and inserted into p*MRPS5*WT‐IRES‐eGFP and p*MRPS5*V336Y‐IRES‐eGFP using MluI‐specific restriction sites.

For generation of stable transfectants, HEK293 cells were transfected with vectors p*MRPS5* V336Y‐IRES‐eGFP and p*MRPS5*WT‐IRES‐eGFP using TurboFect (Thermo Fisher) according to the manufacturer's instructions. Cells were cultured in DMEM supplemented with 10% FBS and propagated under hygromycin B (100 μg/ml) selection for 5–7 weeks. GFP‐expressing colonies were picked for further characterization. GFP fluorescence was analyzed by flow cytometry using the BD FACSCanto II (BD Biosciences) and the FlowJo data analysis software (Tree Star Inc.).

### Determination of *MRPS5* mRNA expression

Quantitative PCR was used to determine *MRPS5* transgene mRNA expression relative to endogenous *MRPS5* mRNA levels. Total RNA was prepared using TRIzol extraction and reverse‐transcribed using the High Capacity RNA‐to‐cDNA Kit (Life Technologies) according to the manufacturer's instructions. A 221‐nt fragment from *MRPS5* cDNA was amplified by using 5′ primers binding specifically either to the endogenous or the transgene *MRPS5* 5′‐UTR (5′‐TGC CCT GGG CGG AGG CCG AGG CGC GGC TC‐3′—forward endogenous gene‐specific; 5′‐GAT CAG AAG CTT CGT TAA CTG AGC TCA GG‐3′—forward transgene‐specific) and a 3′ primer binding to both endogenous and transgene *MRPS5* (5′‐GGC GTA GGG ATG GGT GTC TCT GGT TCC CAG‐3′—reverse). For each sample, SYBR Green qPCR was conducted in triplicate using EvaGreen qPCR Mix (Bio&Sell) and ABI 7500 Fast Real‐Time PCR System (Life Technologies). Amplification consisted of 40 cycles (95°C for 20 s and 60°C for 45 s), and specificity of amplification was confirmed by melting curve analysis. Expression levels of *MRPS5* were quantified as previously described 52.

The ratio of *MRPS5* transgene mRNA to endogenous *MRPS5* mRNA for cell lines expressing mutant V336Y *MRPS5* was determined using *MRPS5* forward 5′‐CTG CCA CAG GGC CAT CAT CAC CAT CTG C‐3′ and *MRPS5* reverse 5′‐CGG AAG AGG CCC TGG GTG AGG CTG AGC‐3′ primers flanking the site of mutation. These primers amplify a 108‐nt PCR fragment of both endogenous and transgene *MRPS5* mRNA. Discrimination was achieved by using TaqMan probes that recognize specifically human wild‐type endogenous *MRPS5* (5′‐CAT GTA TGC CAA GGT C‐3′, conjugated to NED) or human mutant transgene *MRPS5* V336Y (5′‐TAT GCC AAG TAC TCT G‐3′, conjugated to FAM). For each sample, TaqMan qPCR was conducted in triplicate using the TaqMan Kit (Life Technologies) and the ABI 7500 Fast Real‐Time PCR System (Life Technologies). Amplification consisted of 40 cycles (95°C for 20 s and 60°C for 45 s), and the ratio of transgene versus endogenous *MRPS5* was calculated as described previously 52.

### Cellular localization of the transfected MRPS5 protein

For cellular localization of the MRPS5 protein, we introduced a 33‐nt fragment GAG CAA AAG CTC ATT TCT GAA GAG GAC TTG AAT coding for *myc* tag peptide EQKLISEEDLN at the C‐termini of both *MRPS5* WT and *MRPS5* V338Y by PCR, resulting in vectors p*MRPS5*WTmyc‐IRES‐eGFP and p*MRPS5*V336Ymyc‐IRES‐eGFP. HEK293 cells were transiently transfected using TurboFect and cultured in DMEM supplemented with 10% FBS for 72 h. Cellular localization of MRPS5 protein was assessed by Western blot of mitochondrial and cytosolic fractions using anti‐*myc* antibody (ab9106, Abcam).

### Determination of ratio of mitochondrial DNA (mtDNA)/nuclear DNA (nDNA), mitochondrial mass, ATP content, oxygen consumption, and generation time in HEK293 cells

The mtDNA:nDNA ratio was determined as previously described 53. In brief, total DNA was extracted from cultured cells with QIAamp DNA Mini Kit (Qiagen). For mtDNA, a fragment of *MT‐TL1* gene (tRNA leucine) was amplified with forward primer (5′‐CAC CCA AGA ACA GGG TTT GT‐3′) and reverse primer (5′‐TGG CCA TGG GTA TGT TGT TA‐3′), and for nDNA, a fragment of *B2M* gene (beta‐2‐microglobulin) was amplified with forward primer (5′‐TGC TGT CTC CAT GTT TGA TGT ATC T‐3′) and reverse primer (5′‐TCT CTG CTC CCC ACC TCT AAG T‐3′). Amplification was performed in an ABI 7500 real‐time PCR system (Life Technologies) and consisted of 40 cycles (95°C for 20 s, 62°C for 30 s), and specificity of amplification was confirmed by melting curve analysis. Each sample was measured in triplicate.

Mitochondrial mass was measured using MitoTracker Deep Red FM and FACSCanto II (BD Biosciences) equipped with helium–neon laser according to the manufacturer's recommendations. HEK293 cells were seeded in a MULTIWELL™ 12‐well plate (BD Falcon) at a density of 5 × 10^4^ cells per well and cultured overnight in DMEM supplemented with 10% FBS. Cell culture medium was replaced with DMEM containing 200 nM MitoTracker Deep Red FM, and cells were incubated for 30 min at 37°C and 5% CO_2_. Cells were washed twice with PBS and detached from surface with Accutase (Thermo Fisher) and analyzed by FACS.

Total ATP content was quantified using the bioluminescence‐based assay ViaLight Plus Kit (Lonza). 10^4^ cells were seeded in triplicate in a black 96‐well plate (BD Falcon) and incubated for 48 h in 100 μl DMEM supplemented with 10% FBS. 5 μl Alamar Blue (Life Technologies) were added to each well and incubated for 3 h. Alamar Blue was measured as an internal standard, followed by measurement of ATP content according to the manufacturer's protocol.

Mitochondrial respiration was measured using the Seahorse Bioscience XF24 analyzer. XF24 cell culture microplates (Seahorse Bioscience) were coated using 50 mg/ml poly‐D‐lysine, and cells were seeded at a density of 10^5^ cells/well in 100 μl DMEM supplemented with 10% FBS. Cells were incubated for 3 h at 37°C and 5% CO_2_ for attachment, medium was aspirated, replaced by DMEM without FBS, and cells were incubated overnight. Prior to the experiment, the plate was incubated 30 min at 37°C in a CO_2_‐free incubator. Cells were subsequently washed with Mitochondrial Assay Solution (MAS; 70 mM sucrose, 220 mM mannitol, 10 mM KH_2_PO, 4.5 mM MgCl_2_, 2 mM HEPES, 1 mM EGTA, 0.2% (w/v) fatty acid‐free BSA, pH 7.2 at 37°C) according to the manufacturer's protocol (Seahorse Bioscience). Measurements were performed in MAS, supplemented with 10 mM pyruvate, 10 mM succinate, 2 mM malate, and 0.2 nM Plasma Membrane Permeabilizer (PMP) reagent (Seahorse Bioscience). The experiment started in a coupled state in the presence of succinate, pyruvate, and malate (state 2, basal respiration). State 3 was initiated following the injection of 55 μl of 40 mM ADP (4 mM final). State 4o was induced by addition of 62 μl of 25 μg/ml oligomycin (3 μg/ml final). State 3 uncoupled (3u) was assessed after injection of 68 μl 40 μM FCCP (4 μM final) before shutting down mitochondrial respiration by injecting 76 μl of 40 μM and 20 μM (4 μM and 2 μM final) of antimycin A and rotenone, respectively. Drugs were purchased from Seahorse Bioscience. Data were extracted from the Seahorse XF‐24 software, and bioenergetic parameters were calculated according to the manufacturer's guidelines.

Cellular growth was monitored using Alamar Blue (Life Technologies). HEK293 cells were seeded on 24‐well plates (BD Falcon) at low density and incubated in DMEM with 10% FBS at 37°C. 10% Alamar Blue was added to the cells (v/v) at time points 0, 24, 48 and 72 h, and fluorescence was measured (excitation: 530 nm, emission: 590 nm) after 3 h of incubation. Signal intensities at time point 0 h were set as one, and growth curves were plotted. Doubling time was calculated as *t*_D_ = ln(2)/slope.

### Cell‐free MT‐CO1 translation assays

Purified 70S *M. smegmatis* hybrid ribosomes were used in cell‐free translation assays. The mitochondrial *COX1* gene (*MT‐CO1*) was redesigned for efficient bacterial translation: (i) The codon usage was optimized for bacterial translation, (ii) an additional alanine GCG codon was introduced after the ATG start codon to create Kozak context, and (iii) the TAA stop codon was introduced instead of AGA (hungry codon). To measure read‐through, constructs *MT‐CO1*‐AGA‐polyA and *MT‐CO1*‐TGA‐polyA were designed. *MT‐CO1*‐AGA‐polyA has the 69‐nt‐long native 3′ UTR of human *MT‐CO1* mRNA followed by a 51‐nt‐long polyA sequence, *MT‐CO1*‐TGA‐polyA has a TGA stop codon instead of AGA. The constructs were ordered from GenScript USA Inc. and cloned into the pUC57 vector (Thermo Fisher). A typical translation reaction with the total volume of 30 μl contained 0.25 μM 70S ribosomes, 4 μg of *in vitro*‐transcribed mRNA, 40% (vol/vol) of *M. smegmatis* S100 extract, 200 μM amino acid mixture without methionine, cysteine or lysine, 24 units of RiboLock (Thermo Fisher), and 0.4 mg/ml of tRNAs. Energy was supplied by the addition of 12 μl of commercial S30 Premix without amino acids (Promega). Depending on the labeled amino acid, ^35^S‐methionine (KSM‐01; Hartmann Analytic), ^35^S‐cysteine (ARS 0101; Hartmann Analytic), or ^14^C‐lysine (ARS‐0673; Hartmann Analytic) were added; cold methionine, cysteine, or lysine was subsequently added to a final concentration of 0.2 mM. The reaction mixture was incubated at 37°C for 1 h and stopped on ice. To reduce background and for quantification of radioactively labeled bands, the products of the *in vitro* translation reaction were immunoprecipitated. The purified proteins were analyzed on 15% SDS–PAGE. As a control for MT‐CO1, we used *in organello* translation products labeled with ^35^S‐Methionine. The gel was fixed, dried, and exposed on phosphor‐imager screen and scanned with a FLA‐5100 laser scanner (Fujifilm). Intensity of MT‐CO1 bands was quantified using AIDA (version 3.52.046, raytest Isotopenmessgeräte GmbH).

### Mitochondrial *in organello* translation

Mitochondria were isolated from HEK293 cells as described 54. In brief, HEK293 cells were collected from two 90% confluent petri dishes, washed 3 times with PBS, and resuspended in 1 ml of extraction buffer (20 mM HEPES‐KOH (pH 7.5), 0.25 M sucrose, 10 mM KCl, 1.5 mM MgCl_2_, 1 mM EDTA, 1 mM EGTA, 1 mM DTT). Cells were broken by passing ten times through a syringe needle (0.45 × 12 mm), centrifuged 5 min at 800 *g*, and supernatant was transferred to a new tube. The pellet was resuspended in 1 ml of extraction buffer, passed five times through a syringe needle (0.45 × 12 mm), and centrifuged for 5 min at 800 *g*. The supernatant was collected, combined with the previous supernatant, and centrifuged 5 min at 800 *g*. The supernatant was collected and centrifuged for 15 min at 10,000 *g*, and the resulting pellet was used as the mitochondria‐enriched fraction.

Mitochondrial *in organello* translation was done as described, with slight modifications 55, 56, 57. In brief, the mitochondria‐enriched pellet was resuspended in 1 ml of mitochondria reaction buffer (MR‐buffer) containing 20 mM Tris–HCl (pH 7.2), 90 mM KCl, 4 mM MgSO_4_, 1.5 mM KH_2_PO_4_, 20 mM glutamate, 0.5 mM malate, 14 mM sucrose, 44 mM sorbitol, 4 mM ADP, 0.1 mM amino acids (without methionine and cysteine), 1 mg/ml BSA (fatty acids free), and 0.1 mg/ml cycloheximide. Depending on the labeled amino acid, 15 μl of ^35^S‐methionine or ^35^S‐cysteine was added. Cold methionine or cysteine was subsequently added to a final concentration of 0.1 mM. The resulting master mix was split into 100 μl per translation reaction. *In organello* translation was conducted by incubation at 30°C for 2 h on a shaker (800 rpm). After incubation, the reaction mixtures were centrifuged for 5 min at 15,000 *g*, and the supernatants were discarded. The pellets were washed with cold washing buffer containing 10 mM Tris–HCl (pH 7.4), 320 mM sucrose, 1 mM EDTA, centrifuged for 5 min at 15,000 *g*, and resuspended in 20 μl of 1× SDS loading buffer or in 30 μl H_2_O for subsequent immunoprecipitation. The samples were analyzed on 15% SDS–PAGE. The gel was fixed, dried, and exposed on phosphor‐imager screen and scanned with laser scanner FLA‐5100. Intensity of bands was quantified using AIDA.

### Immunoprecipitation

In brief, for one reaction 50 μl of Dynabeads M‐280 sheep anti‐mouse IgG (11202D, Thermo Fisher) or sheep anti‐rabbit IgG (11204D, Thermo Fisher) was washed 3 times with 1 ml of blocking solution (PBS with 0.5% BSA). For immobilization of antibodies, Dynabeads were incubated with 5 μg of monoclonal MT‐CO1 antibody (ab14705, Abcam), monoclonal MT‐CO2 antibody (ab110258, Abcam), or polyclonal antibody against Lys_14_ (customized antibody, GenScript USA Inc.) in 1 ml of blocking solution overnight at 4°C, subsequently washed three times with blocking solution, and resuspended in 50 μl of blocking solution.

To 30 μl of bacterial or *in organello* translation reaction, 20 μl of 20× IP buffer (0.4 M Tris–HCl, pH 7.5, 1.5 M NaCl, 0.5% SDS, 10% Triton X‐100, 10 mM EDTA) was added, incubated for 30 min at room temperature, and H_2_O was added to a final volume of 350 μl. 50 μl of Dynabeads with the immobilized antibodies was added to the translation mixture and incubated overnight at 4°C while shaking. The Dynabeads were washed with blocking solution, pelleted, and resuspended in 20 μl of H_2_O. 6 μl of 4× SDS loading buffer was added and vortexed. The samples were heated at 95°C for 5 min and resolved on 15% SDS–PAGE. The gel was fixed, dried, and exposed on phosphor‐imager screen and scanned with laser scanner FLA‐5100. Intensity of MT‐CO1 and MT‐CO2 bands was quantified using AIDA. For validation of antibodies used, see Fig 2F and G.

### MT‐CO1 translation in rabbit reticulocyte lysate

*MT‐CO1*‐AGA‐polyA mRNA and *MT‐CO1*‐TGA‐polyA mRNA were used for translation in rabbit reticulocyte lysates. A typical translation reaction with a total volume of 30 μl contained 20 μl of rabbit reticulocyte lysate (Promega), 4 μg of *in vitro*‐transcribed mRNA, 200 μM amino acid mixture without methionine, 24 units of RiboLock (Thermo Fisher) and 1 μl of ^35^S‐methionine (KSM‐01; Hartmann Analytic). The reaction mixture was incubated at 37°C for 1 h and stopped on ice. The products of the *in vitro* translation reaction were immunoprecipitated using MT‐CO1 or poly‐lysine antibodies. The immunoprecipitated proteins were analyzed on 15% SDS–PAGE. As a control for MT‐CO1, we used *in organello* translation products labeled with ^35^S‐methionine. The gel was fixed, dried, and exposed on a phosphor‐imager screen and scanned with a FLA‐5100 laser scanner (Fujifilm).

### Mitochondrial translation

HEK293 cells were grown in 10‐cm petri dishes (BD Falcon) in DMEM with 10% FBS to 80% confluence, washed, and incubated for 30 min in DMEM without methionine. Media were exchanged to DMEM without methionine supplemented with 100 μM emetine (Sigma‐Aldrich) and incubated for 15 min. The medium was replaced with 500 μl methionine‐free DMEM supplemented with 2 μl of ^35^S‐methionine and 100 μM emetine, and cells were incubated for 2 h at 37°C. The supernatant was carefully aspirated, and cells were detached by pipetting with 1 ml of PBS. The cells were washed 2 times with PBS and resuspended in 200 μl of 1× SDS loading buffer. The samples were analyzed on a 15% SDS–PAGE. The gel was fixed, dried, and exposed on a phosphor‐imager screen and scanned with the FLA‐5100 laser scanner. A pilot experiment was performed to control that emetine effectively inhibited cytosolic protein synthesis and that the residual protein synthesis reflects mitochondrial translation as shown by susceptibility to linezolid (Pfizer) (Appendix Fig S3G).

### Western Blot

Cells were grown to 70% confluence in DMEM with 10% FBS. Cells were lysed with 1× Passive Lysis Buffer (Promega) and ultrasonicated. Lysates were centrifuged (15,000 *g*, 10 min), and protein concentration in the supernatant was measured by the Micro BCA Protein Assay Kit (Thermo Fisher). Ten micrograms of total protein was resolved on 10% SDS–PAGE and blotted on nitrocellulose membranes, which were probed with specific antibodies. Amersham ECL Prime Western blotting detection reagent (RPN2232; GE Healthcare) was used as substrate for horseradish peroxidase (HRP). The specific antibodies used in this study were as follows: anti‐myc tag (ab9106, Abcam); anti HSP60 (ab46798, Abcam), anti‐ATP6 (MS508, MitoScience), and anti‐β‐tubulin (ab15568, Abcam); HRP‐conjugated goat anti‐rabbit (G‐21234, Thermo Fisher); and goat anti‐mouse antibodies (A10551, Thermo Fisher).

### Generation of Knock‐In *Mrps5* transgenic mice

*Mrps5* V338Y mice were generated using a FLEx approach 58. This strategy is based on the duplication of the exon to be mutated and its insertion in the antisense orientation. The duplicated regions are flanked with *loxP* and *lox511* cloned in opposite directions (*Mrps5* ^FLEx^ allele). Upon Cre induction, the mutated exon will be flipped resulting in the deletion of the wild‐type exon and expression of the mutant protein (*Mrps5* ^V338Y^ allele).

The gene‐targeting vectors were constructed from genomic 129Sv mouse‐strain DNA. For *Mrps5* V338Y, exon 10 was duplicated and the mutant exon inserted in antisense within intron 10. The insertion points were selected in order not to disrupt any potential regulatory region identified by consensus sequences. The duplicated exons were flanked by loxP and mutated lox511 sites for deletion of the WT exon and switch of the mutant exon upon Cre‐mediated action. A Flippase Recognition Target (FRT)‐flanked neomycin cassette was cloned into the targeting vector. Linearized targeting vectors were transfected into 129Sv ES cells, and positive selection was started 48 h after electroporation by addition of G418. Resistant clones were isolated, amplified, duplicated, and genotyped by both PCR and Southern blot analysis. The following primer pairs were designed to specifically amplify the targeted locus—34690sa: 5′‐GGG AAC TTC CTG ACT AGG GGA GGA GTA G‐3′, 34691sa: 5′‐GCA AAG ACT CAG ACA AAC AAC CGA CG‐3′. Targeted locus was confirmed by Southern blot analysis using internal and external probes on both 5′ and 3′ ends. PCR and Southern blot genotyping led to the identification of two targeted clones for *Mrps5* V338Y.

Recombined ES cell clones were microinjected into C57BL/6 blastocysts and gave rise to male chimeras. Breeding with C57BL/6 Flp deleter mice (CAG‐Flp) was performed to produce the *Mrps5* heterozygous inducible line devoid of neomycin cassette: *Mrps5* ^FLEx/WT^. Inducible mice were backcrossed to C57BL/6 for four generations. The inducible *Mrps5* V338Y line was crossed to C57BL/6 CRE deleter mice (CMV‐Cre) to produce the *Mrps5* V338Y heterozygous induced mutant line. Cre‐recombination resulted in flipping of the duplicated inverted exon bearing the mutation of interest (FLEx strategy) and expression of the mutant allele, resulting in heterozygous mutant line *Mrps5* ^V338Y/WT^ referred to as induced mice. For each line, heterozygous mice were genotyped by PCR, Southern blot, and sequencing (Appendix Fig S4).

Heterozygous inducible mice (*Mrps5* ^FLEx/WT^) were first identified by PCR using primer 017 (5′‐GCC AAA GAG ACA TGC AGT GAG AAG AGT ACC‐3′) and primer 018 (5′‐CCA CCA TGA GTC CAA TGA TTG CAC C‐3′). The primers result in amplification of a specific 1.0 kb fragment for the *Mrps5* ^FLEx^ allele, while wild‐type *Mrps5* does not result in an amplicon. PCR‐positive mice (*Mrps5* ^FLEx/WT^ *)* were confirmed by Southern blot analysis using a 3′ external probe (amplified using the following primers: 5′‐ACT TTA CAG CCT AGC TCG TCA GCA CAG C‐3′, 5′‐CCC AAG CTA AGA CCC CTT TCA ACA GC‐3′). Upon digestion with NheI, a 5.1 kb fragment is expected for the *Mrps5* ^WT^ allele, and a 5.5 kb fragment is expected for the *Mrps5* ^FLEx^ allele. Heterozygous induced mice (*Mrps5* ^V338Y/WT^) were genotyped by PCR using primers 34712hom (5′‐GCT TCT GTT TGT GGC TTG TGT TGC C‐3′) and 34713hom (5′‐TTG TTG GAC TGG TGA AAC ACC T CGG‐3′). The primers result in a 0.6 kb gene fragment for the *Mrps5* ^WT^ allele, and a 0.8 kb gene fragment for the *Mrps5* ^V338Y^ allele. Heterozygous induced mice (*Mrps5* ^V338Y/WT^) were confirmed by Southern blot analysis using a 5′ external probe (amplified using the following primers: 5′‐GGT GGC TTT TGC AGT AAT TTT ATT TACT TGC‐3′, 5′‐AAA CTC CCT AAG CTT CTC CCC ACA CTC‐3′). Following digestion with AvrII/HpaI, the following fragment sizes are expected for each allele: 9.6 kb for *Mrps5* ^WT^, 7.9 kb for *Mrps5* ^FLEx^, and 7.2 kb for *Mrps5* ^V338Y^. The presence of the mutation in the induced mice (*Mrps5* ^V338Y/WT^) was controlled by sequencing of a 2.8 kb product specific for the induced allele using primers hybridizing to the *lox*P site (remaining upstream of exon 10 upon induction) and downstream of exon 11. The primers used (primer 017: 5′‐GCC AAA GAG ACA TGC AGT GAG AAG AGT ACC‐3′, primer 34709cof: 5′‐ATG AGG GGA GGG GCA GGA ACC TAA‐3′) will not amplify the wild‐type allele.

Mice tissue sections were frozen until RNA extraction was performed. RNA was extracted using TRIzol as described above. cDNA synthesis was performed using SuperScript II cDNA Synthesis Kit (Life Technologies) using random hexamers and 40 ng RNA in 20 μl reagent according to the manufacturer's protocol. Expression of the mutant gene was assessed from brain, inner ear, and retina recovered from 6‐ to 7‐week‐old animals. Heterozygous *Mrsp5*‐induced mice (*Mrps5* ^V338Y/WT^) were compared to wild‐type mice for expression of the *Mrps5* gene. RT–qPCR analysis showed no significant difference in *Mrps5* total mRNA between heterozygous induced mutant mice and controls. Expression of the V338Y mutant allele was demonstrated by sequencing of the RT–PCR product. Primers used for RT–PCR and sequencing were EBO4‐SeqF1 (5′‐GCT ATT GGG AAA GCT GCT GA‐3′) and EBO4‐SeqR2 (5′‐TCA CGT CCT GCC AGT CCA GC‐3′). Primers used for RT–qPCR analysis were EBO4‐TOT‐F1 (5′‐CCA TGA ACA TGC TCA ACC TC‐3′) and EBO4‐TOT‐R2 (5′‐ATA GGC AGA GGC CCA CAT T‐3′).

Heterozygous *Mrps5* ^V338Y/WT^ mice are viable, are fertile, and do not show any gross phenotype. Heterozygous mutant mice bred within the expected Mendelian ratio, suggesting that the mutation has no significant effect on the viability of the line. Interbreeding *Mrps5* ^V338Y/WT^ resulted in homozygous *Mrps5* ^V338Y/V338Y^ mice. Analysis of *Mrps5* gene expression in the homozygous mutant mice revealed *Mrps5* mutant mRNA only in all tissues tested (Appendix Fig S4). The overall expression level of mutant *Mrps5* mRNA was comparable to wild‐type mice, indicating that the mutation did not lead to deregulation of *Mrps5* gene expression. Homozygous mutant mice were able to breed and did not show any gross phenotype.

### RNA sequencing and data analysis

RNA sequencing (RNA‐seq) was performed at the UZH/ETH Functional Genomics Center Zurich (FGCZ) according to the Illumina RNA sequencing protocol. For transcriptome analysis, four clones of *Mrps5* V336Y and six clones of *Mrps5* wild‐type transfected cells were compared. RNA was extracted using TRIzol. The quality of the isolated RNA was determined using a Qubit^®^ (1.0) Fluorometer (Life Technologies) and a Bioanalyzer 2100 (Agilent). Only those samples with a 260 nm/280 nm ratio between 1.8 and 2.1 and a 28S/18S ratio within 1.5‐2 were further processed. The TruSeq Stranded mRNA Sample Prep Kit (Illumina) was used in the succeeding steps. Briefly, total RNA samples (100–1,000 ng) were ribosome‐depleted and then reverse‐transcribed into double‐stranded cDNA with actinomycin added during first‐strand synthesis. The cDNA samples were fragmented, end‐repaired, and polyadenylated. TruSeq adapters containing the index for multiplexing were ligated to the fragmented DNA samples. Fragments containing TruSeq adapters on both ends were selectively enriched with PCR. The quality and quantity of the enriched libraries were validated using the Qubit^®^ (1.0) Fluorometer and LabChip^®^ GX (Caliper Life Sciences). The product is a smear with an average fragment size of approximately 360 bp. The libraries were normalized to 10 nM in Tris‐Cl 10 mM, pH 8.5 with 0.1% Tween‐20. The TruSeq SR Cluster Kit v4‐cBot‐HS (Illumina) was used for cluster generation using 8 pM of pooled normalized libraries on the cBOT. Sequencing was performed on the Illumina HiSeq 2500 single end 126 bp using the TruSeq SBS Kit v4‐HS (Illumina). The quality of the reads was assessed using FastQC (Babraham Bioinformatics), and potential contaminations were evaluated with FastQ Screen (Babraham Bioinformatics) using bowtie2 v. 2.1.0 default parameters 59. Quantification of gene expression was performed using the RSEM package (version 1.2.18) 60 mapping against the ensembl 75 annotations derived from the human genome assembly GRCh37 and mouse genome assembly GRCm37. Genes not present (< 10 counts per gene) in at least 50% of samples from one condition were discarded from further analyses. Differential gene expression analysis between sample groups of interest was performed using the R/bioconductor package edgeR 61. To evaluate functional activities, differentially expressed genes were mapped to known biological ontologies based on the GO project using gene annotation tool Enrichr 62, 63.

Transcriptome data are available in Gene Expression Omnibus (GEO), accession no. GSE101503.

### Histopathological analyses

Mutant *Mrps5* ^V338Y/V338Y^ mice of 9 months of age were histologically analyzed in comparison with age‐matched wild‐type littermates (three animals per group). 4‐μm sections of formalin‐fixed, paraffin‐embedded (FFPE) brain and cerebellum were subjected to hematoxylin–eosin (H&E) staining. In addition, FFPE brain sections were Holmes–Luxol‐stained (to assess axon integrity and myelination status) and processed for immunohistochemistry (IHC) using anti‐GFAP from DAKO (1:500) and anti‐Iba1 from Wako (1:3,000); secondary antibody was anti‐rabbit IgG (Vector; ImmPRESS Reagent Kit).

### Cortical brain homogenate preparation

Isolated cortical hemispheres were dissected on ice, washed in ice‐cold buffer 1 (138 mM NaCl, 5.4 mM KCl, 0.17 mM Na_2_HPO_4_, 0.22 mM K_2_HPO_4_, 5.5 mM glucose, 58.4 mM sucrose, pH 7.35), and homogenized with a glass homogenizer (10–15 strokes, 400 rpm) in 2 ml of ice‐cold buffer 2 (210 mM mannitol, 70 mM sucrose, 10 mM HEPES, 1 mM EDTA, 0.45% BSA, 0.5 mM DTT, 5× Complete Protease Inhibitor (Roche Diagnostics).

### Preparation of isolated mitochondria

Cortical brain homogenates were centrifuged at 1,450 *g* for 7 min at 4°C to remove nuclei and tissue particles; centrifugation was repeated with the supernatant fraction for 3 min 64. The resulting supernatant fraction was centrifuged at 10,000 *g* for 5 min at 4°C to pellet mitochondria. The resulting pellet was resuspended in 1 ml of ice‐cold buffer 2 and centrifuged at 1,450 *g* for 3 min at 4°C to remove debris. The mitochondria‐enriched supernatant was centrifuged at 10,000 *g* for 5 min at 4°C to obtain the mitochondrial fraction. This fraction was resuspended in 300 μl of PBS and stored at 4°C until use, followed by determination of protein content.

### Oxygen consumption and ATP measurements in isolated brain mitochondria

Rates of oxygen consumption were measured in isolated cortical mitochondria using a Seahorse Bioscience XF24 Analyzer, following the manufacturer's protocol and as previously described 64. Briefly, mitochondria were diluted 1:10 in cold 1× MAS containing 10 mM succinate, 2 mM malate, and 10 mM pyruvate. 50 μl of mitochondrial suspension (5 μg mitochondrial protein/well) was delivered to each well of a XF Cell Culture microplate (except for background correction wells) and centrifuged at 2,000 *g* for 20 min at 4°C to let mitochondria adhere to the wells. After centrifugation, 450 μl of pre‐warmed (37°C) 1× MAS plus substrates was added to each well and the plate was incubated 5 min at 37°C in a CO_2_‐free incubator prior to the experiment. The plate was placed in a XF24 Analyzer, and oxygen consumption rates were assessed under different respiratory states as described above for HEK293 cells, except that oligomycin was added to a final concentration of 2.5 μg/ml.

ATP content was determined using ViaLight Plus Kit (Lonza) following the manufacturer's instructions and normalization per protein content.

### Complex IV and complex II measurements in isolated brain mitochondria

Mitochondrial complex IV activity was determined in intact isolated mitochondria (1 μg) using the Cytochrome c Oxidase Assay Kit (Sigma) as previously described 65. The colorimetric assay is based on the observation that a decrease in absorbance at 550 nm of ferrocytochrome c is caused by its oxidation to ferricytochrome c by cytochrome c oxidase. Briefly, the assay was performed in a 96‐well plate. Isolated mitochondria were diluted in enzyme dilution buffer (10 mM Tris–HCl, 250 mM sucrose, pH 7) to a final concentration of 0.2 μg/μl. 5 μl of the mitochondrial solution was added per well. Subsequently, 195 μl of assay buffer (10 mM Tris–HCl, 120 mM KCl, pH 7) was added, then 10 μl of ferrocytochrome c was injected in each well, and the absorbance at 550 nm was measured for 60 s using a Victor X5 multi‐wavelength reader (PerkinElmer).

Mitochondrial complex II activity was determined in intact isolated mitochondria using complex II‐mediated reduction of 2,6‐dichlorophenolindophenol (DCIP). Isolated mitochondria were placed in 1 ml of medium containing 60 mM KH_2_PO_4_ (pH 7.4), 3 mM KCN, 20 μg/ml rotenone, 20 mM succinate, and 5 μg mitochondrial protein. The reaction was initiated by the addition of 1.3 mM phenazine methosulfate (PMS) and 0.18 mM DCIP. Reduction of DCIP was followed by measuring the decreasing absorbance at 600 nm. The extinction coefficient used for DCIP was 21 mM/cm. Complex IV and II activities were normalized as per mg protein content.

### Determination of superoxide anion radicals

MitoSOX™ Red reagent (Thermo Fisher) is a fluorogenic dye specifically targeted to mitochondria in live cells. Oxidation of MitoSOX™ Red reagent by superoxide produces red fluorescence. Cortical brain homogenate samples were adjusted to 1 mg protein/ml in HBSS. 150 μl of a 5 μM MitoSOX™ reagent working solution (prepared according to the manufacturer's protocol) was added to 250 μl sample, followed by incubation at 37°C for 10 min, protected from light. Then, samples were centrifuged for 3 min at 500 *g*. After discarding the supernatant, the pellets were washed three times with 250 μl HBSS (3 min at 500 *g*). Finally, the samples were transferred into a 96‐well plate (final volume 100 μl per well) and fluorescence was detected using the Victor X5 multiplate reader at 510 nm (excitation) and 580 nm (emission). The intensity of fluorescence is proportional to superoxide anion radicals in mitochondria.

### Noise exposure and auditory brain stem response

Male C57BL/6 mice were first exposed for 2 h to broadband noise (2–10 kHz) at intensities of 104, 106, 108, and 110 dB. ABR thresholds were determined 3 weeks later when damage to the auditory system has stabilized and is no longer confounded by transient impairment of sound processing. The frequencies of 12, 24, and 32 kHz were selected for measurement to cover the mid‐frequency to high‐frequency regions of the cochlea. Exposures at noise intensities of 108 and 110 dB resulted in robust elevations of ABR thresholds and were chosen as treatment for mutant *Mrps5* ^V338Y/V338Y^ mice and littermate controls. Sham‐exposed controls (2 h in the exposure chamber without sound) showed no effect on ABR.

### Animals for behavioral analysis

Mice were housed under a 12/12‐h light–dark cycle (lights on at 20:00) in groups of 2–5, unless individual housing was required by experimental protocols. Mice were tested during the dark phase of the cycle under indirect dim light (ca. 12 lux) unless specified otherwise. Before behavioral testing, each mouse was tagged by subcutaneous injection of a RFID microchip (Planet ID GmbH) under brief isoflurane inhalation anesthesia. Three cohorts comprising a total of 34 female mice were examined in the same sequence of behavioral tests (in the order listed below) starting at the age of approximately 9 and 19 months, and selected tests were also recorded at 14 months.

### IntelliCage system

The IntelliCage apparatus (TSE Systems) was placed in a polycarbonate cage (20.5 cm high, 58 × 40 cm top, 55 × 37.5 cm bottom, Techniplast, 2000P) and accommodated up to 16 mice 66. A food rack provided standard mouse chow *ad libitum*. Woodchip bedding as well as four standard red shelters were provided. Four triangular conditioning chambers (15 × 15 × 21 cm) were fitted in the cage corners. Two drinking bottles were accessible in each of them behind motorized doors. Visits, nosepokes, and licks were recorded and assigned to individual mice through a RFID antenna at the chamber entrance. A PC running IntelliCage Plus software (TSE Systems) collected the data and controlled the experiments. Testing began by free adaptation (7 days) providing access to all bottles with doors open at all times. During subsequent nosepoke adaptation (3–7 days), doors were closed but could be opened at any time by a nosepoke. Finally, drinking session adaptation (4–7 days) shaped mice to drink only during 4 1‐h drinking sessions each day. The following learning tasks were based on the same drinking session schedule 67. In all of them, percentage of rewarded visits with nosepokes served as measure of performance. In the corner‐preference task, each mouse could obtain water only in one of the four corners during 4 days. In the corner‐preference serial reversal task (7 days), the mice had to learn a new corner during every drinking session. During the final chaining task (10 days), the mice had to visit corners in a clock or anti‐clockwise sequence to get water.

### Individual home cage activity

Diurnal home cage activity of individually housed mice was recorded during 7 days under the standard light cycle using a rack of type II long mouse cages (365 mm long × 207 mm wide × 140 mm high) equipped with one passive IR sensor per mouse (ActiviScope, New Behavior Inc) 68.

### Video tracking

During open field and water‐maze tests, on the elevated O‐maze, and during the light–dark transition test, the mice were video‐tracked using a Noldus EthoVision 3.00 system (Noldus Information Technology). The system recorded position, object area, and the status of defined event recorder keys on the keyboard. Raw data were then transferred to public domain software Wintrack 2.4 (http://www.dpwolfer.ch/wintrack) for further analysis.

### Open field

The round arena had a diameter of 150 and 35 cm high sidewalls made of white polypropylene 68. Each subject was released near the wall and observed for 10 min on two subsequent days. Vertical activity was estimated by counting reductions in object size in the absence of locomotion.

### Elevated O‐maze

A 5.5‐cm‐wide annular runway with an outer diameter of 46 cm was positioned 40 cm above the floor 68. Two opposing 90° closed sectors were protected by inner and outer walls (height 16 cm). The remaining two open sectors had no walls. Animals were released near a closed sector and observed for 10 min. Head dips were recorded using the keyboard event recorder. Head dips that occurred while the animal kept its body between the protection walls were classified as protected head dips.

### Grip test

The grip meter consisted of a mechanical newton meter (max. force: 300 g) that was positioned horizontally 9 cm above a wooden plate and attached to a metallic T‐bar of 5 cm width and 3 mm thickness. Mice were held by the tail and allowed to grasp the bar with both forepaws. They were then gently pulled away until they released the bar. Five consecutive measurements were obtained each during two sessions with an interval of approximately 1 h 69.

### Light–dark transition test

The apparatus consisted of a transparent chamber (20 × 30 cm, 20 cm high) exposed to direct room light (ca. 450 lux) and connected to an enclosed dark box (20 × 15 cm, 20 cm high) via a small opening 68. Mice were released in the bright compartment and observed for 5 min.

### Water‐maze place navigation

The round white polypropylene pool had a diameter of 150 cm with 68 cm high walls. It was filled with water (24–26°C, depth 15 cm), which was rendered opaque by addition of 1 l of milk (UHT whole milk 3.5% fat, Coop). The white quadratic goal platform (14 × 14 cm) was made of metallic wire mesh and painted white. It was hidden 0.5 cm below the water surface in the center of one of the four quadrants, approximately 30 cm from the side wall. Salient extra‐maze cues were placed on the walls of the testing room. Animals performed 30 training trials (max. duration 120 s), 6 per day with intertrial intervals of 30–60 min and varying starting positions. During the first 18 trials, the hidden platform was held in the same position (acquisition phase) and then moved to the opposite quadrant for the remaining 12 trials (reversal phase). The first 60 s of the first reversal trial served as probe trial to test for spatial retention.

### Nesting test

Mice were provided with 3 g of compressed cotton (Plexx) in a single cage and given 24 h to build a nest. Nests were assessed on a rating scale of 1–5 70.

### Burrowing test

A gray plastic tube (inner diameter 6.3 cm, length 18.2 cm) with one open end was filled with 350 g of standard diet food pellets and placed at a slight angle into a large standard transparent type III cage. Each mouse was given 24 h to remove pellets from the tube 71.

### T‐maze spontaneous alternation

Arms of the T‐shaped maze measured 30 × 10 cm with 20 cm high side walls. Goal and start arms could be blocked with manually operated doors. Three trials each was run on two subsequent days with an interval of about 60 min 72. During the sample phase, the animal was allowed to choose a goal arm and was confined to the chosen goal arm for 30 s. After this, the animal was transferred back to the start arm and again allowed to choose one of the goal arms (choice phase).

### Acoustic startle response

The SR‐LAB™ SDI startle response system (San Diego Instruments) was controlled by proprietary SR‐LAB software. The cylindrical animal enclosure (28 × 89 mm) was made of clear Perspex, rested on a plate with a motion sensor and was enclosed in a sound‐attenuated ventilated cabinet. The loudspeaker was located above the animal and produced white noise pulses. To record a startle response profile, 66 trials were presented in total after a habituation time of 300 s. Nine different sound levels (dB) used were as follows: 64, 68, 72, 76, 80, 90, 100, 110, and 120. Each stimulus was 40 ms and presented 6 times in pseudorandom order such that each sound level occurred once within a block of nine trials. The series began and ended with six presentations each of the 120 dB stimulus. The average intertrial interval was 15 s (ranging from 10 to 20 s). The startle response was recorded for 250 ms (measuring the response every 1 ms) starting with the onset of the startle stimulus. Background noise was set at 64 dB during the entire session.

### Hotplate test

To assess acute thermal nociception, the incremental hot/cold plate analgesia meter (model PE34, IITC Life Science) was set to 55°C. The animal was observed until it licked a hindpaw, jumped, or until the cutoff time (60 s) was reached.

### Water‐maze cue navigation

Testing apparatus and procedures were the same as used for the place navigation task, except that the platform was marked with a salient local cue and moved to a new position for every trial. Each mouse was trained for 2 days with six trials each.

### Statistical analysis

Data from biochemical assays were analyzed with Prism 5.0 software using unpaired Student's *t*‐test to determine the significance of difference unless otherwise specified.

Data from behavioral studies were analyzed using an ANOVA model with genotype (*Mrps5* ^V338Y/V338Y^, *Mrps5* ^WT/WT^) and age cohort (9 months, 19 months) as between‐subject factors. Within‐subject factors were added as needed to explore the dependence of genotype effects on place, time, or stimulus. Interactions were further explored by pairwise *t*‐tests or by splitting the ANOVA model, as appropriate. Variables with strongly skewed distributions or strong correlations between variances and group means were subjected to Box‐Cox transformation before statistical analysis. The false discovery rate (FDR) control procedure of Hochberg was applied to groups of conceptually related variables within single tests to correct significance thresholds for multiple comparisons. Similarly, FDR correction was applied to *t*‐tests during post hoc testing. Statistical analyses and graphs were produced using R version 3.2.3, complemented with the packages ggplot2, psych, and moments. All tests were two‐sided with significance set at *P* < 0.05.

### Ethics statement

Animal experiments were approved by the Veterinary Office of the Canton of Zurich (licenses 29/2012 and 44/2015). For ABR measurements, animal care was supervised by the University of Michigan's Unit for Laboratory Animal Medicine and experimental protocols were approved by the University of Michigan Committee on Use and Care of Animals (protocol no. PRO00006852).

## Data availability

RNA‐seq data are available under Gene Expression Omnibus (GEO) GSE101503.

## Author contributions

Concept and design of study, coordination, and supervision of experiments: ECB; MT‐CO1 and MT‐CO2 assays: RA and PF; modeling, generation of stable transfectants, luciferase misreading, and read‐through assays: DS and SD; analysis of stable transfectants: DS, SD, RA, RJ, YT, and HB; behavioral studies: A‐KF and DW; construction and generation of transgenic mice: PI‐P and KT; analysis of brain cortical mitochondria: KS and AE; ABR studies: NO and JS; transcriptome studies: RJ and HR; histopathology studies: BO and SF; writing of the manuscript with input from all authors: ECB, EW, SD, JS, and DW. All authors analyzed and discussed the data.

## Conflict of interest

The authors declare that they have no conflict of interest.

## Supporting information

Appendix

Review Process File
