# Supplementary material for: Mutant MRPS5 affects mitoribosomal accuracy and confers stress‐related behavioral alterations
Source: EMBO Rep. 2018 Sep 20;19(11):e46193. doi: 10.15252/embr.201846193 (PMC6216279; doi:10.15252/embr.201846193)
Supplement: Supplementary file 1 — Appendix [file EMBR-19-e46193-s001.pdf]

Mutant MRPS5 affects mitoribosomal accuracy and confers stress-related behavioral alterations

Rashid Akbergenov, Stefan Duscha, Ann-Kristina Fritz, Reda Juskeviciene, Naoki Oishi, Karen Schmitt, Dimitri Shcherbakov, Youjin Teo, Heithem Boukari, Pietro Freihofer, Patricia Isnard-Petit, Björn Oettinghaus, Stephan Frank, Kader Thiam, Hubert Rehrauer, Eric Westhof, Jochen Schacht, Anne Eckert, David Wolfer, Erik C. Böttger

Appendix items

- **p. 1 Table of contents**
- **p. 2 Appendix Figure S1**
- **p. 3 Appendix Figure S2**
- **p. 4 Appendix Figure S3**
- **p. 5 Appendix Figure S4**
- **p. 6 Appendix Figure S5**
- **p. 7-11 Appendix Figures Legends**
- **p. 12 Appendix Table S1**

Appendix Figure S1

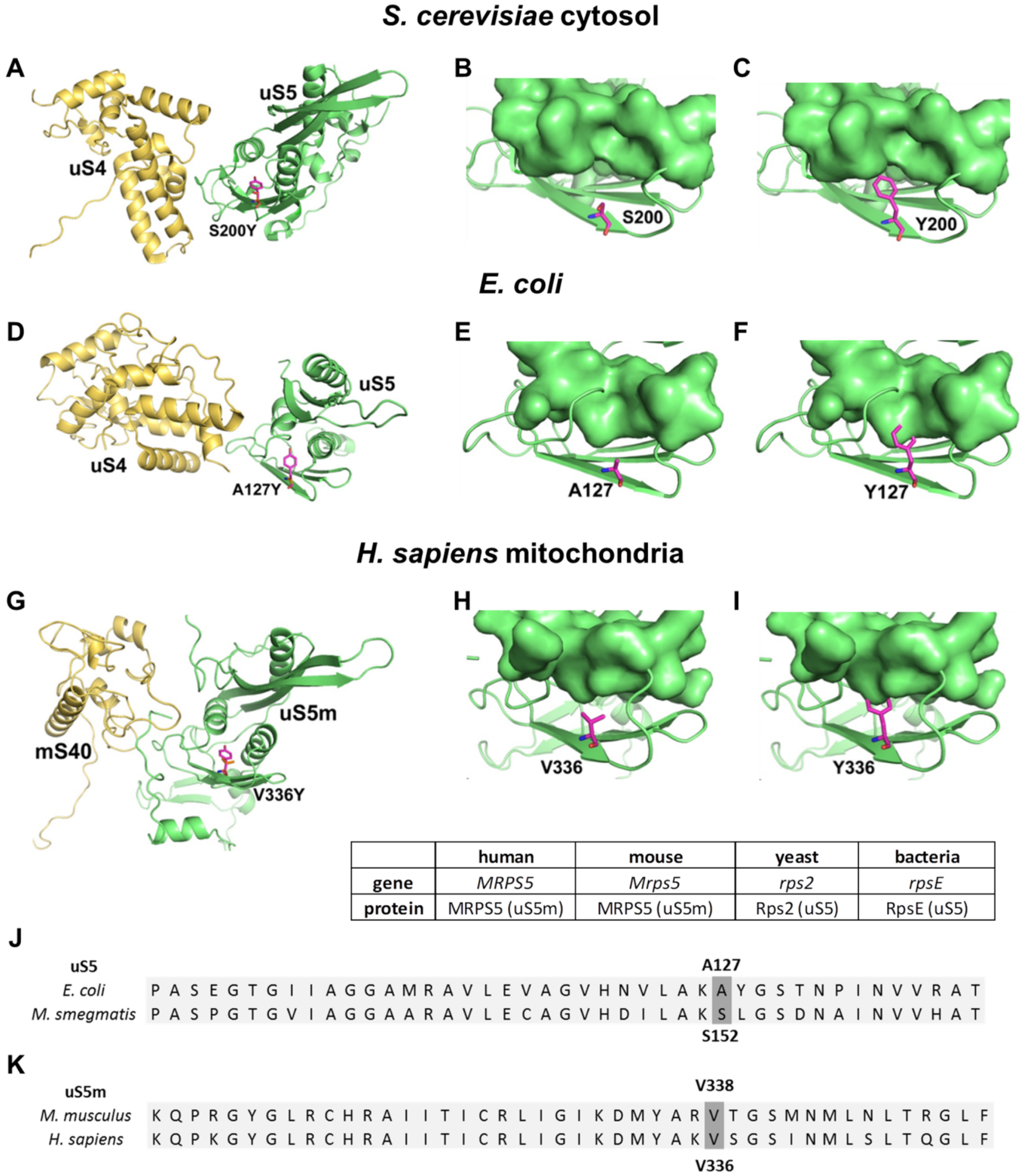

	human	mouse	yeast	bacteria
**gene**	*MRPS5*	*Mrps5*	*rps2*	*rpsE*
**protein**	MRPS5 (uS5m)	MRPS5 (uS5m)	Rps2 (uS5)	RpsE (uS5)

J

uS5 — **A127** (*E. coli*), **S152** (*M. smegmatis*)

E. coli	P A S E G T G I I A G G A M R A V L E V A G V H N V L A K **A** Y G S T N P I N V V R A T
M. smegmatis	P A S P G T G V I A G G A A R A V L E C A G V H D I L A K **S** L G S D N A I N V V H A T

K

uS5m — **V338** (*M. musculus*), **V336** (*H. sapiens*)

M. musculus	K Q P R G Y G L R C H R A I I T I C R L I G I K D M Y A R **V** T G S M N M L N L T R G L F
H. sapiens	K Q P K G Y G L R C H R A I I T I C R L I G I K D M Y A K **V** S G S I N M L S L T Q G L F

Appendix Figure S2

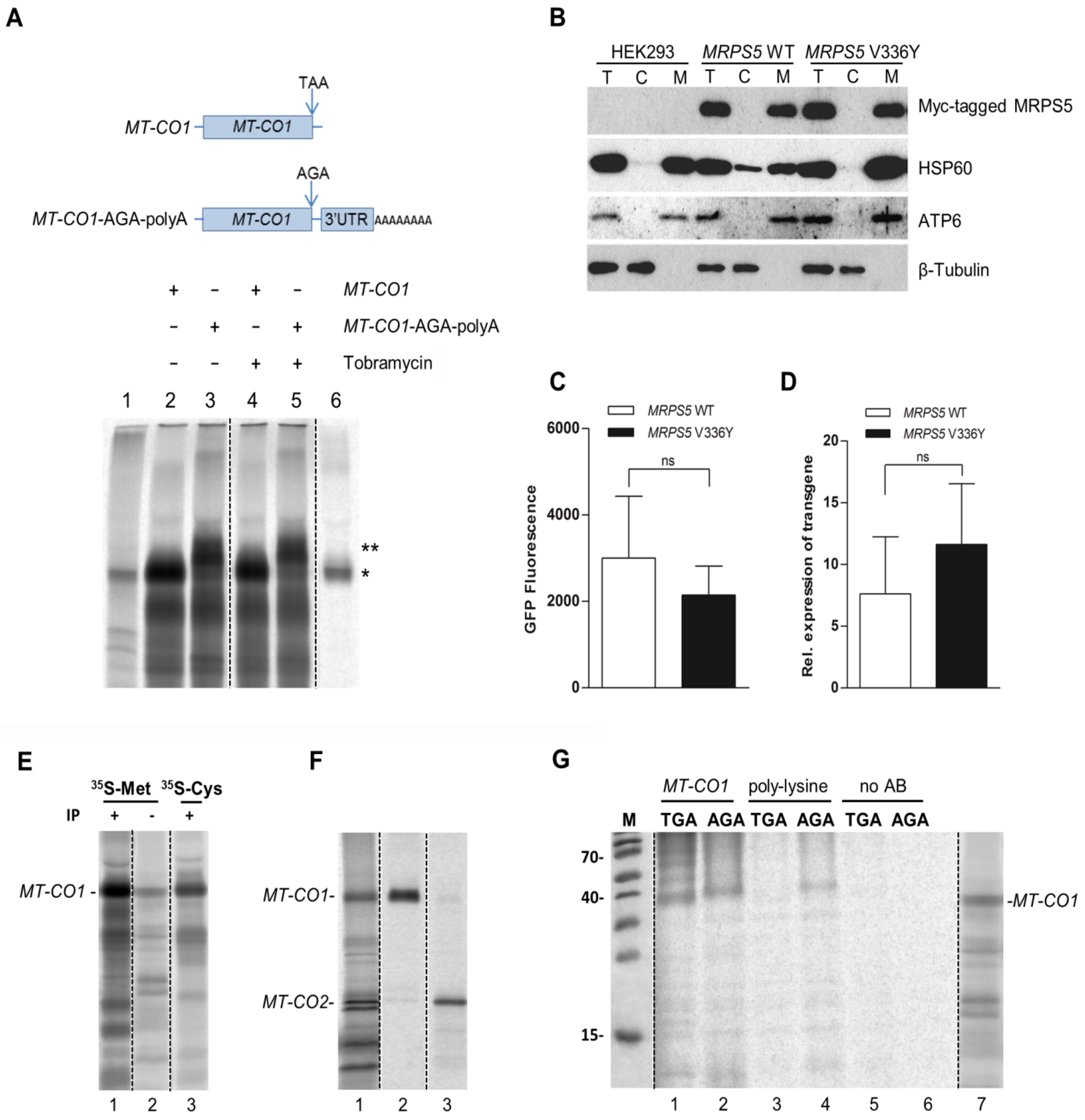

Appendix Figure S3

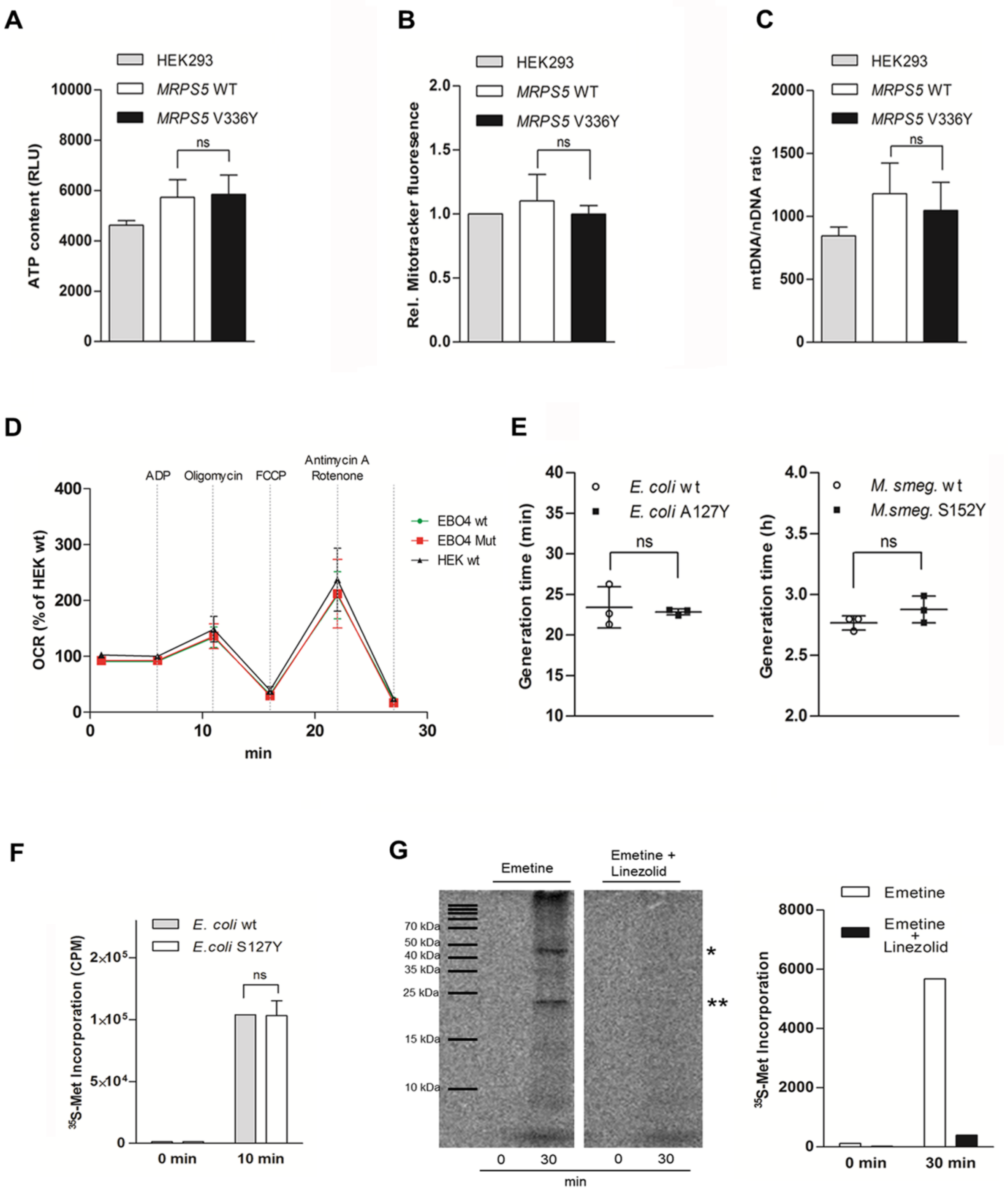

Appendix Figure S4

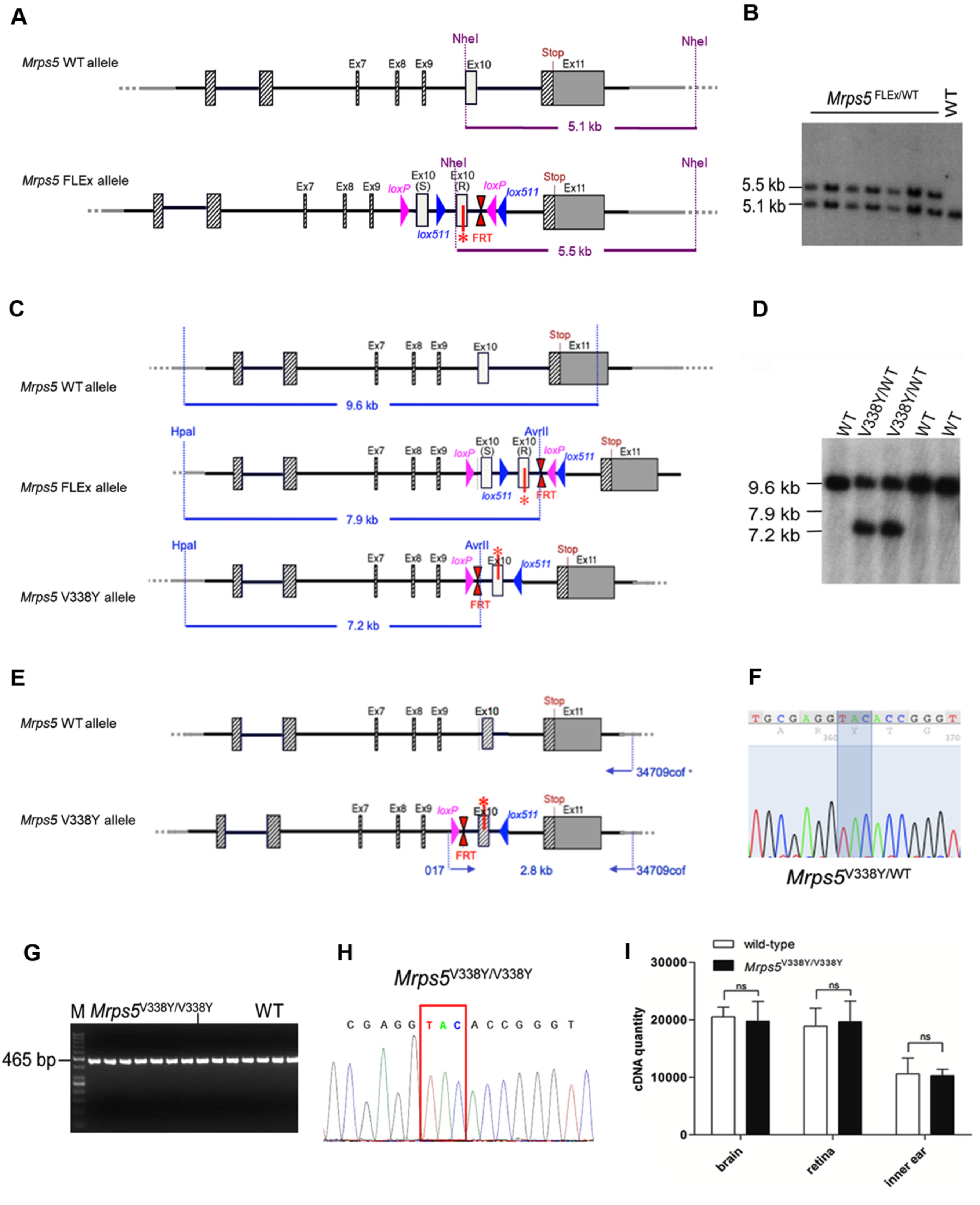

Appendix Figure S5

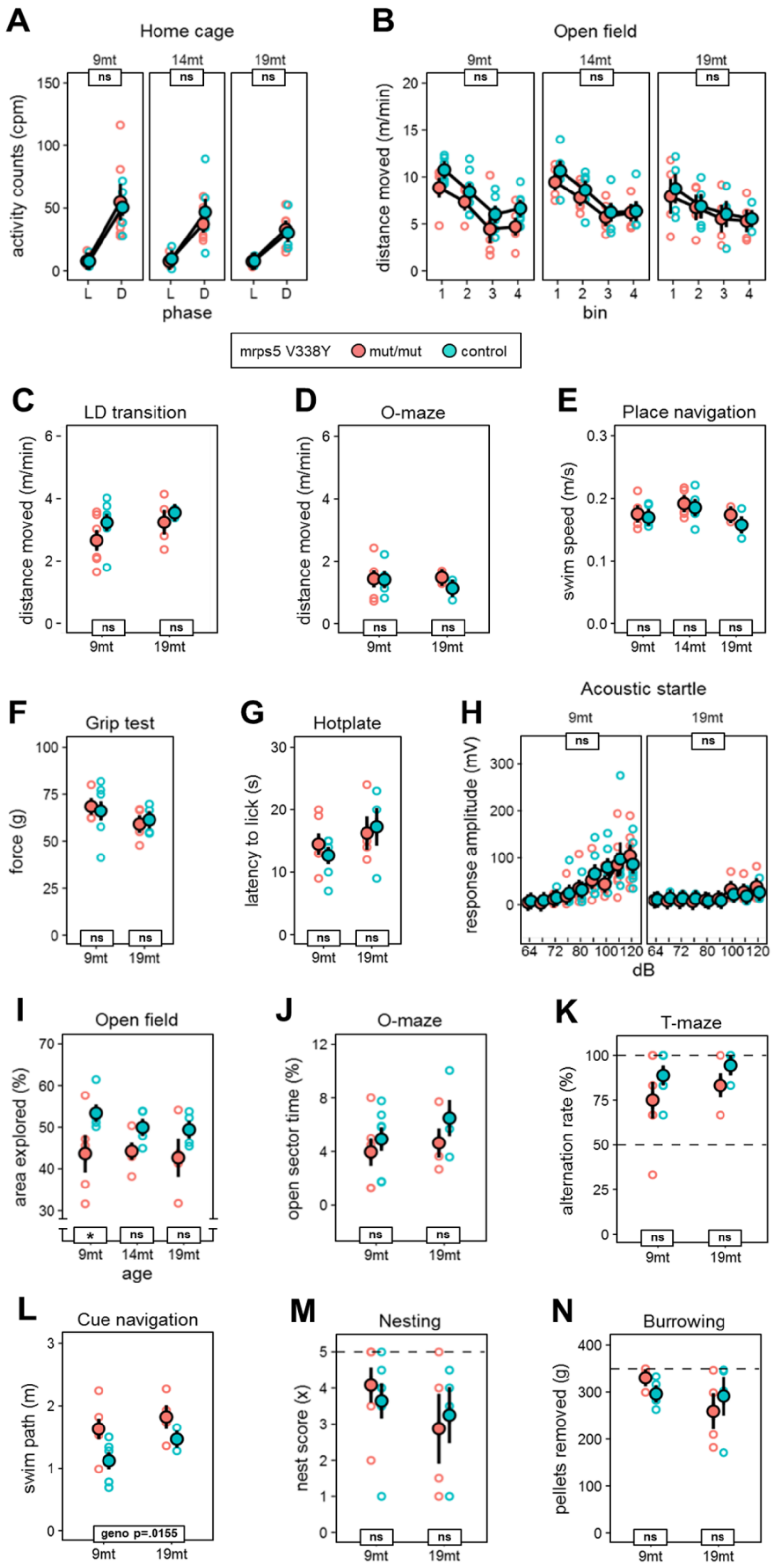

Appendix Figure Legends

Appendix Figure S1 – Modelling amino acid substitutions in ribosomal protein uS5.

Visualization was done using PyMol v.1.8 (Schrödinger, Inc.) on the basis of available crystal structures.

A Protein-protein interface uS4 (Rps9)-uS5 (Rps2) in the *S. cerevisiae* cytosolic ribosome; uS4 and uS5 are shown in gold and green, respectively.

B, C Sterical hindrance within the C-terminal domain of uS5 resulting from S200Y mutation; for better visualization the α-helices are shown as surface (Source: PDB 4V7R).

D Protein-protein interface uS4 (RpsD)-uS5 (RpsE) in the *E. coli* ribosome. uS4 and uS5 are shown in gold and green, respectively. Residue A127 and mutation A127Y are marked in magenta.

E, F Demonstrate sterical hindrance within the C-terminal domain of uS5 for the A127Y mutation (Source: PDB 4YBB).

G Protein-protein interface mS40-uS5m (MRPS5) in the human mitochondrial ribosome; mS40 and uS5m are shown in gold and green, respectively. Mitochondria-specific ribosomal protein mS40 structurally replaces uS4 in the mitoribosome.

H, I Sterical hindrance within C-terminal domain of uS5m resulting from V336Y mutation (Source: PDB 3J9M).

J Sequence alignment of *E. coli* and *M. smegmatis* ribosomal protein uS5 (RpsE). The mutated amino acid position (homolog of *S. cerevisiae* uS5 S200) is indicated – *E. coli* numbering A127, *M. smegmatis* numbering S152.

K Sequence alignment of *M. musculus* and *H. sapiens* uS5m (MRPS5). The mutated amino acid position (homolog of *S. cerevisiae* uS5 S200) is indicated – *H. sapiens* numbering V336, *M. musculus* numbering V338. The nomenclature and spelling for ribosomal proteins and its encoding genes is given in the box [73].

Appendix Figure S2

A Autoradiography of proteins immunoprecipitated with MT-CO1 Ab. *In-vitro* translation reactions of mitochondrial mutant A1555G hybrid ribosomes using *MT-CO1* mRNA, *MT-CO1*-AGA-polyA mRNA, and ^{35}S-Met labelling (lanes 2-5). Tobramycin was used at 5 µM. Lanes 1 and 6 are controls. Lane 1: ^{35}S-Met labelled *in-organello* translation reaction; lane 6: MT-CO1 Ab immunoprecipitation of ^{35}S-Met labelled *in-organello* translation. * indicates MT-CO1, **

indicates MT-CO1 extended version. Schemed *MT-CO1* and *MT-CO1*-AGA-polyA constructs used for *in-vitro* translation are given on top.

B Western blot to determine cellular localization of myc-tagged MRPS5 protein. HEK293 cells were transiently transfected using *MRPS5* WT or *MRPS5* V336Y constructs; cell lysates were fractionated (T - total, C – cytosolic fraction, M - mitochondrial fraction). Myc-tag antibody (Ab) to detect MRPS5, HSP60 Ab as marker of proteins translated in the cytosol but localized in mitochondria, ATP6 Ab as marker for proteins translated and localized in mitochondria, β-tubulin as marker for proteins translated and localized in the cytosol.

C Mean GFP fluorescence of HEK cells stably transfected with *MRPS5* WT or *MRPS5* V336Y (n=8 clones each, + SD); ns, not significant (Student's t-test).

D Relative mRNA expression of *MRPS5* transgene in comparison to endogenous *MRPS5* in stably transfected HEK293 cells determined by qPCR (n=8 clones, + SD); ns, not significant (Student's t-test).

E Autoradiography of proteins immunoprecipitated with MT-CO1 Ab. *In-vitro* translation reactions using mutant A1555G hybrid ribosomes and *MT-CO1* mRNA. Lane 1: ^{35}S-Met labelled *in-vitro* translation reaction; lane 3: ^{35}S-Cys labelled *in-vitro* translation. Representative figure used for quantification of ^{35}S-Cysteine and ^{35}S-Methionine ratio. Lane 2: *In-organello* translation reaction labelled with ^{35}S-Met used as control for MT-CO1.

F Validation of MT-CO1 and MT-CO2 antibodies used for immunoprecipitation. Lane 1: Autoradiography of *in-organello* mitochondrial translation labelled with ^{35}S-Met; lane 2: Immunoprecipitation of *in-organello* translation products with MT-CO1 antibody; lane 3: Immunoprecipitation of *in-organello* translation products with MT-CO2 activity. Each antibody used immunoprecipitated the corresponding protein.

G Validation of poly-lysine antibodies used for immunoprecipitation. *In-vitro* translation reactions using rabbit reticulocytes lysates, *MT-CO1*-TGA-polyA mRNA, *MT-CO1*-AGA-poly mRNA, and labelling with ^{35}S-Met. Following *in-vitro* translation, proteins were immunoprecipitated with MT-CO1 (lanes 1 and 2) and poly-lysine antibodies (lanes 3 and 4). As a control, beads without immobilized antibodies were used (lanes 5 and 6). *In-organello* mitochondrial translation products derived from HEK293 wild-type cells labelled with ^{35}S-Met were used as control for MT-CO1 (lane 7). The poly-lysine antibody immunoprecipitated the MT-CO1 protein with an AGA arginine codon and a 3' poly-lysine tail, but not the MT-CO1 protein with a TGA stop codon. For scheme of mRNA constructs used, see Fig. 1F.

Appendix Fig S3

A ATP content in HEK293 cells, *MRPS5* WT and *MRPS5* V336Y HEK cells (n=7 clones each, + SD); ns, not significant (Student's t-test).

B Determination of mitochondrial mass using Mitotracker Red in HEK293 cells, *MRPS5* WT and *MRPS5* V336Y HEK293 cells (n=8 clones each, + SD); ns, not significant (Student's t-test).

C Measurement of mtDNA/nDNA ratio by qPCR in HEK293 cells, *MRPS5* WT and *MRPS5* V336Y HEK293 cells (n=8 clones, + SD); ns, not significant (Student's t-test).

D Oxygen consumption rate (OCR) in HEK293, MRPS5 WT and MRPS5 V336Y cell lines. Data were normalized to basal respiration in HEK293 cells, set to 100% ($n_{MRPS5\ WT}$=4, $n_{MRPS5\ V336Y}$=5, ± SEM).

E Generation time of *E. coli* WT and A127Y mutant (left panel, n=3), generation time of *M. smegmatis* WT and S152Y mutant (right panel, n=3); ns, not significant (Student's t-test).

F Translation efficiency in *E. coli* WT and S152Y mutant assessed by ^{35}S-Met incorporation (n=3, + SD); ns, not significant (Student's t-test).

G Ribosomal inhibition by emetine and linezolid selectively blocks cytosolic versus mitochondrial translation. Left: Autoradiography of emetine (100 µM) treated HEK293 cells after 0 and 30 min of incubation with ^{35}S-Met versus emetine (100 µM) and linezolid (1 mM) treated HEK293 cells after 0 and 30 min of incubation with ^{35}S-Met; emetine was used to inhibit cytosolic translation and linezolid was used to inhibit mitochondrial translation. * and ** indicate MT-CO1 and MT-CO2 bands, respectively. Right: Quantitative analysis of ^{35}S-Met incorporation by densitometric analysis (n=2).

Appendix Fig S4 – Generation of *Mrps5* mutant mice.

A Southern blot strategy for detection of *Mrps5*^{FLEx} alleles. Schematic representation of wild-type and *Mrps5*^{FLEx} allele, restriction sites used for Southern blot are indicated.

B Representative example of Southern blot analysis. The genomic DNA of seven mice was compared to wild-type DNA (WT). NheI digested DNA was blotted on nylon membrane and hybridized with the external 3' probe indicated.

C Southern blot strategy for detection of heterozygous induced mutant *Mrps5*$^{V338Y/WT}$ Knock-in mice. Schematic Southern blot representation of inducible mutant *Mrps5* Knock-in allele, induced mutant *Mrps5* Knock-in allele, and *Mrps5* wild-type allele.

D Representative example of Southern blot genotyping. Southern blot was performed using double AvrII/HpaI digestion of genomic DNA and hybridization with the external 5' probe indicated to detect inducible and induced mutant *Mrps5* allele and *Mrps5* wild-type allele, WT controls are indicated.

E Presence of the point mutation in induced mutant *Mrps5* Knock-in mice. Schematic representation of the PCR strategy, primers are specific for the induced mutant *Mrps5* Knock-in allele.

F Representative DNA sequencing result of *Mrps5*$^{V338Y/WT}$ induced mice, using genomic DNA as template.

G Representative RT-PCR result of *Mrps5*$^{V338Y/V338Y}$ mice and WT mice.

H Representative sequencing result of *Mrps5*$^{V338Y/V338Y}$ RT-PCR amplicon.

I Mean values quantification (cDNA quantity) of *Mrps5* transcripts in brain, inner ear and retina from *Mrps5*$^{V338Y/V338Y}$ mice and wild-type mice (n=5-6 animals, mean values + SD of cDNA quantity for wild-type and *Mrps5*$^{V338Y/V338Y}$ mice are represented, ns, not significant, Student's t-test).

Appendix Fig S5

A-D Activity in familiar and novel environments.

E-H Sensory-motor function.

I, J Exploration and anxiety.

K Alternation rate in the T-maze.

L Cue navigation in the water-maze.

M, N Species-typical behaviors.

Graphs show mean and ± SEM. Post-hoc FDR corrected ns $p \geq 0.10$, t-test for genotype within age cohorts. 34 mice, n=17 per genotype, n=9-13 per age cohort.

A Normal diurnal change of activity in the home cage (ANOVA phase $F_{1,28}=412.8$ $p<0.0001$).

B Distance moved in the open field recorded in bins of 5 min (ANOVA: bin $F_{3,72}=46.19$ $p<0.0001$).

C Light-dark transition test, distance moved while in the bright compartment.

D Elevated O-maze, distance moved.

E Average swim speed during the water-maze place navigation task.

F Grip force, average of 10 trials.

G Hot plate test, latency to lick hind paw.

H Acoustic startle response as function of stimulus intensity (64, 68, 72, 76, 80, 90, 100, 110, 120 dB) (ANOVA: stimulus $F_{2,32}=3.315$ $p=0.0491$, age $F_{1,16}=16.63$ $p=0.0009$).

I Open field, % of areas explored (ANOVA: genotype $F_{1,25}=11.51$ $p=0.0024$, Post-hoc *$p<0.05$).

J Elevated O-maze, % protected head dips.

K T-maze, % alternation averaged across 6 trial pairs.

L Water-maze cue navigation, swim path averaged across 12 training trials (ANOVA: $F_{1,15}=7.448$ $p=0.0155$).

M Nesting test, next quality score after 24 h.

N Burrowing test, pellets removed after 24 h.

Appendix Table S1 – Cognate and near-cognate codons for cysteine and methionine in genes coding for MT-CO1 and MT-CO2 proteins.

		COX1	COX2
**Methionine** tRNA anti-codon **CAU**	**Cognate**	**32**	**10**
	AUA	25	8
	AUG	7	2
	**Near-cognate**	**48**	**26**
	CUG	4	3
	GUG	3	0
	UUG	0	1
	AAG	1	0
	ACG	2	0
	AGG	0	0
	AUC	26	15
	AUU	12	7
**Cysteine** tRNA anti-codon **GCA**	**Cognate**	**1**	**3**
	UGU	0	1
	UGC	1	2
	**Near-cognate**	**96**	**29**
	CGC	2	1
	AGC	4	0
	GGC	17	4
	UUC	29	7
	UCC	10	4
	UAC	18	9
	UGA	16	3
	UGG	0	1
